# Association between infants’ serum levels of 26 metals and gut microbiota: a hospital-based cross-sectional study in China

**DOI:** 10.3389/fmicb.2025.1669475

**Published:** 2025-12-11

**Authors:** Xing Yan, Jun Qiu, Ruiwen Huang, Xiaoming Peng, Shi-ting Xiang, Kunyan Zhao, Yunlong Peng, Yan Zhuang, Ye Ma, Mingyang Wu, Fei Yang

**Affiliations:** 1 Xiangya School of Public Health, Central South University, Changsha, China; 2 Pediatrics Research Institute of Hunan Province, The Affiliated Children’s Hospital of Xiangya School of Medicine, Central South University (Hunan Children’s Hospital), Changsha, China; 3 Department of Neonatology, The Affiliated Children’s Hospital of Xiangya School of Medicine, Central South University (Hunan Children’s Hospital), Changsha, China; 4 Hunan Province Key Laboratory of Typical Environmental Pollution and Health Hazards, School of Public Health, Hengyang Medical School, University of South China, Hengyang, China; 5 Department of Epidemiology and Health Statistics, Medical College of Soochow University, Suzhou, China

**Keywords:** infants, metals mixture, MaAslin2, rare earth metals, gut microbiota

## Abstract

**Background:**

This study examined the associations of toxic metals, essential metals, and rare earth elements with infant gut microbiota at Hunan Children’s Hospital, China.

**Methods:**

Generalized linear regression (GLR) was used to assess individual metal associations with alpha diversity, whereas Bayesian kernel machine regression (BKMR) and weighted quantile sum (WQS) regression were applied to evaluate metal mixture-taxa relationships.

**Results:**

Results showed that barium (Ba) and arsenic (As) were positively associated with the Chao1 index, whereas chromium (Cr), antimony (Sb), tungsten (W), cobalt (Co), copper (Cu), lanthanum (La), praseodymium (Pr), and uranium (U) showed negative associations. Six antagonistic interactions were identified: Cr-W (β = −2.57), Cr-La (β = −3.82), Tl-As (β = −4.48), As-La (β = −4.31), As-Pr (β = −5.85), and La-Pr (β = −2.38). Two synergistic interactions were observed: Sb-Pr (β = 2.17) and Sb-U (β = 2.14). BKMR analysis identified Mn as a key contributor to *Burkholderia-Caballeronia-Paraburkholderia* abundance (PIP = 0.535). Metal mixture exposure was positively linked to *Ralstonia* abundance, with As having the highest contribution (PIP = 0.886). Cu was the primary driver of *Clostridium_ sensu_stricto_1* abundance (PIP = 0.867), with synergistic Mn-Cu (β = 0.797) and Ba-Cu (β = 0.720) interactions.

**Discussion:**

These findings demonstrate that As and Cu are the most influential metals on gut microbial alpha diversity, whereas Cu, As, and Mn significantly influence specific microbial taxa, providing novel epidemiological evidence on metal-gut microbiota interactions in vulnerable infants.

## 1 Introduction

Infants are colonized by microbes at birth and gradually stabilize to an adult-like community structure after 3 years of age (Yatsunenko et al., 2012; Korpela and de Vos, 2018). Early life microbial colonization shapes metabolism and immunity (Charbonneau et al., 2016; Gensollen et al., 2016; Robertson et al., 2019; Donald and Finlay, 2023), and the disruption of optimal microbial succession may contribute to lifelong and intergenerational deficits in growth and development (Robertson et al., 2019). Sufficient microbial exposure is essential for proper immune development in early life (Donald and Finlay, 2023). Dysbiosis during this period is linked to diseases in children and adults, including autism, attention deficit hyperactivity disorder, allergies, and asthma (Gensollen et al., 2016; Tamburini et al., 2016; Ronan et al., 2021).

Previous studies have indicated that host genetics, prenatal environment, and delivery mode can influence the newborn microbiome at birth. This initial community is subsequently modulated by gestational age and postnatal factors, such as antibiotic exposure, diet, or environmental exposure (Tamburini et al., 2016; Robertson et al., 2019; Vandenplas et al., 2020). Metals and metalloids cause serious environmental pollution in China (Schmid and Xiong, 2023). These elements exist in almost all types of environmental media (Li et al., 2022; Peng et al., 2022). Breast milk, inhalation of air, or skin contact with contaminated soil, air, or dust are the main ways for children to contact metals (Li et al., 2022; Yan et al., 2022). Previous studies have investigated the association between certain metals/metalloids and the gut microbiota of infants (Eggers et al., 2019; Laue et al., 2020; Sitarik et al., 2020; Shen et al., 2022; Zeng et al., 2022; Xiang et al., 2024), but the results are inconsistent, and research on metal mixture exposure remains limited. Recent studies have shown the joint effects of metal mixture exposure on adverse pregnancy and birth outcomes (Savabieasfahani et al., 2020; Wei et al., 2020; Howe et al., 2021; Liu et al., 2021; Hu et al., 2022; Liu J. et al., 2022; Ovadia et al., 2023; Issah et al., 2024), suggesting that the current research should consider scenarios of combined metal exposure rather than focusing solely on individual metals. Furthermore, an in-depth investigation into the effects of early life multi-metal exposure on the gut microbiota could yield mechanistic explanations for the biological impacts mediated by metal mixtures. Although our team previously conducted a study on toxic metal exposure and gut microbiota in neonates admitted to the neonatal intensive care unit (NICU) (Xiang et al., 2024), it had several limitations, including a small sample size, narrow scope of metals analyzed, and unexamined metal–metal interactions. Notably, a significant knowledge gap persists regarding the relationship between the infant gut microbiome and exposure to rare earth elements, radioactive metals, and metal mixtures.

To address these gaps, this study systematically examined the association between 26 metal elements (including rare earth elements and radioactive metals) and gut microbiota in infants recruited from a hospital in Hunan, China. By employing multiple mixture exposure assessment methods, this study elucidates the combined effects of metal mixtures on the gut microbiota and explores potential interactions among metal elements. These findings provide valuable epidemiological evidence and offer scientific insights for developing health protection strategies for vulnerable pediatric populations.

## 2 Materials and methods

### 2.1 Study population and design

This was a hospital-based cross-sectional study conducted at Hunan Children’s Hospital in China from 1 August 2018 to 31 October 2019. With the approval of the Medical Ethics Committee of Hunan Children’s Hospital (HCHL-2018-64) and written informed consent signed by their parents, 459 newborns were recruited for this study (Xiang et al., 2024). Maternal pregnancy and newborn characteristics were collected via face-to-face interviews with parents, and neonatal hospitalization data were obtained from the medical record system. Blood samples used for exposure assessment were collected in inert separation tubes during the first venipuncture for admission tests. Serum was separated immediately after collection and stored at −80 °C. The first fecal defecation after hospitalization was collected from each infant, immediately placed on ice, transported to the laboratory within 2 h, and stored at −80 °C until DNA extraction. The median age of the newborns at the time of fecal and serum sample collection was 23 and 21 days, respectively. Of the initial 459 infants, 342 were ultimately included in this study.

### 2.2 Metal measurement

26 metals/metalloids were measured, including chromium (Cr), manganese (Mn), nickel (Ni), cadmium (Cd), tin (Sn), antimony (Sb), cesium (Cs), barium (Ba), tungsten (W), mercury (Hg), thallium (Tl), lead (Pb), iron (Fe), cobalt (Co), copper (Cu), zinc (Zn), arsenic (As), selenium (Se), rubidium (Rb), strontium (Sr), molybdenum (Mo), lanthanum (La), cerium (Ce), praseodymium (Pr), thorium (Th), and uranium (U). The limits of detection (LOD) are provided in the Supplementary Table S2. These elements were selected because of their environmental relevance to Hunan Province’s rich mineral resources (Chen et al., 2023; Hu et al., 2022; Jiang et al., 2018) and their high detection rates in children (Liang et al., 2024; Shen et al., 2022; Tan et al., 2022), enabling a comprehensive assessment of their effects on the gut microbiota. Specific methodologies for metal measurements are provided in the Supplementary materials. The concentrations of metals below the LOD were imputed as the LOD divided by the square root of 2.

### 2.3 DNA extraction and sequencing

16S rRNA amplicon sequencing was performed by Genesky Biotechnologies Inc., Shanghai, China. Bacterial DNA was extracted from fecal samples using the QIAamp DNA Stool Mini Kit according to the manufacturer’s protocol. The concentration and purity of genomic DNA were detected using a Nanodrop 2000 and Qubit 3.0 Spectrophotometer, and the integrity was detected by agarose gel electrophoresis. The isolated bacterial DNA was used as a template for amplifying the V4-V5 region of the 16S rRNA gene with the primers 515F (5’-GTGCCAGCMGCCGCGG-3′) and 907R (5’-CCGTCAATTCMTTTRAGTTT-3′). Sequencing of 16S rRNA was performed on an Illumina NovaSeq 6,000 platform to generate 2 × 250 bp paired-end reads. Raw read sequences were processed in QIIME2, and adapter and primer sequences were trimmed using the Cutadapt plug-in.

The DADA2 plugin was used for quality control and identification of amplicon sequence variations (ASVs). A pre-trained Naive Bayes classifier trained on RDP (version 11.5) was used to obtain taxonomic assignments of ASV representative sequences with a confidence threshold of 0.8.

### 2.4 Covariates

Seven previously identified covariates associated with the gut microbiota were included as potential confounders: sex (male/female), antibiotic exposure (no/yes), ever breastfed (no/yes), delivery mode (vaginal/cesarean), birth weight (continuous), preterm (gestational age≥37 weeks is considered full term, <37 and ≥32 weeks is preterm, < 32 weeks is very preterm), and age at sampling (continuous). Generalized linear regression (GLR) models were used to estimate the associations between individual covariates and alpha-diversity indices (Supplementary Table S2).

### 2.5 Statistical analysis

The distributions of the demographic characteristics and 26 serum metals were summarized using descriptive statistics. Population characteristics are presented as mean (SD) for continuous variables and number (%) for categorical variables. Correlations among metal concentrations were tested using Spearman’s correlation analysis.

Alpha diversity was assessed using the Chao1 (community richness), Shannon and Simpson (richness and evenness), and Pielou (evenness) indices, which were computed using the vegan package in R. The metal concentrations were log-transformed to approximate normality before analysis.

To determine the most predictive metals for children’s gut microbiota alpha diversity, we applied elastic net regression (ENR), incorporating all the measured metals. The optimal penalty parameter (*λ*) was selected via 10-fold cross-validation by minimizing the mean squared error (MSE) (Liu C. et al., 2022). Additionally, GLR models (Xiang et al., 2024) were employed to evaluate the associations between individual metals and alpha-diversity indices, adjusting for covariates. By combining these findings, we identified metals that significantly influenced alpha diversity, which were then used for the mixture effect analysis.

Beta diversity (between-subject) was assessed based on the Bray-Curtis distance. Permutational multivariate analysis of variance (PERMANOVA) was performed with the adonis function in the R package “vegan” to estimate the statistical significance of the association of individual metal concentrations to beta-diversity (Anderson, 2017), while adjusting for the aforementioned covariates.

The multivariate analysis method (MaAsLin2) was used to determine the relationship between metals and taxa. All taxa data were normalized to the relative abundance before screening (Mallick et al., 2021). To facilitate subsequent BKMR analysis, a pseudo count (half of the lowest non-zero relative abundance value for each taxon) was added to the zero-count data prior to normalization and log2 transformation. The analysis was conducted using default parameters. Taxa (including phyla and families) were included for screening if they were present in more than 10% of participants (Shen et al., 2022). Taxa were regressed against individual metal measurements with adjustments for the predefined set of covariates. Associations were selected from the raw MaAsLin2 output based on individual metals and their corresponding taxa, and statistical significance was determined using FDR-adjusted q-values.

Bayesian kernel machine regression (BKMR) with variable selection was implemented to model the associations between metals and MaAsLin2-identified metal-associated genera (Bobb et al., 2014; Laue et al., 2020; Xiang et al., 2024). The Markov chain Monte Carlo algorithm was used to achieve 10,000 iterations of variable selection. The importance of the variables was quantified by calculating their posterior inclusion probabilities (PIPs). Elements selected for inclusion in more than 50% of the iterations [PIP > 0.5] were deemed to be significant contributors to the variability in the outcome. Potential metal–metal interactions identified by BKMR were further examined by incorporating interaction terms into generalized linear models, with significance assessed via *p*-values.

Weighted quantile sum (WQS) regression was employed to assess the overall effect of metal mixtures on microbial taxa previously identified by Maaslin2. The analysis was conducted using the R package “gWQS,” which empirically constructs a WQS index as a weighted sum of individual metal concentrations. The dataset was randomly divided into a training set (40%) for weight estimation and a validation set (60%) for statistical inference. Within the training set, 1,000 bootstrap samples were generated to robustly estimate the weight of each metal. A positive constraint was applied to the model, assuming a unidirectional overall mixture effect, and weights were averaged over 100 repeated holdout validation runs to improve the stability of the estimates. The resulting WQS index (ranging from 0 to 1) represents the combined exposure level of the metal mixture, with metals exhibiting non-negligible weights identified as components of concern. The final estimate was interpreted as the change in microbial taxon abundance associated with a one-quantile increase in all metal concentrations simultaneously (Carrico et al., 2015; Chen et al., 2022). BKMR and WQS models were also used to evaluate the combined effects of metals on alpha diversity. All models are adjusted for preterm status, sex, antibiotic exposure, ever breastfed, delivery mode, birth weight, and age at sampling.

To assess the potential effect-modifying role of preterm birth, stratified analyses were conducted by categorizing the participants into full-term (≥37 weeks), preterm (<37 weeks), and very preterm (<32 weeks) subgroups. The same analytical methods were applied independently for each stratum. All statistical analyses were performed using R 4.3.1, with multiple comparisons addressed via Benjamini–Hochberg false discovery rate correction (significance threshold: *q* < 0.1).

## 3 Results

### 3.1 Study participant characteristics

This study analyzed a cohort of 342 infants hospitalized because of premature birth or health conditions. The cohort comprised 212 (62%) males, with a mean age of 26.50 ± 22.83 days (Table 1). Most infants (177 [51.8%]) were delivered by cesarean section, and the average birth weight was 2339.75 ± 785.96 g, with a gestational age of 34.71 ± 3.36 weeks. Additionally, 196 (57.3%) were not breastfed, and 254 (74.3%) had antibiotic exposure. All metals were detected in over 75% of the samples (Supplementary Table S2).

The distributions of the metals are presented in Supplementary Table S2. Several rare metals (La, Ce, Pr, and Th) were highly correlated (Spearman’s correlation coefficient > 0.7; Supplementary Figure S2).

**Table 1:** Characteristics of the 342 infants [*N*, (%) or mean (± SD)].

Characteristics	*N*, (%) or mean (SD)
Birth weight (g)	2339.75 (785.96)
Gestational age (week)	34.71 (3.36)
Age at sampling (day)	26.50 (22.83)
Sex	
Male	212 (62%)
Female	130 (38%)
Ever breastfed	
No	196 (57.3%)
Yes	46 (42.7%)
Delivery mode	
Vaginal Delivery	165 (48.2%)
Cesarean	177 (51.8%)
Antibiotic exposure	
No	88 (25.7%)
Yes	254 (74.3%)

### 3.2 Alpha diversity

Elastic net regression (ENR) analysis (Supplementary Figure S1) identified Tl as the key predictor for both Shannon and Simpson indices. For the Chao1 index, Cd, Sb, Ba, W, Co, Cu, As, and U were selected as predictors, whereas Cs and Tl were selected as significant predictors for the Pielou index. Notably, the effect sizes of the predictive elements were relatively small in the ENR analysis. However, generalized linear models (GLMs) revealed a different pattern: (1) neither Tl nor Cs showed significant associations with Shannon, Simpson, or Pielou indices, and (2) Cd exhibited no significant relationship (Supplementary Table S3).

**Figure 1:**
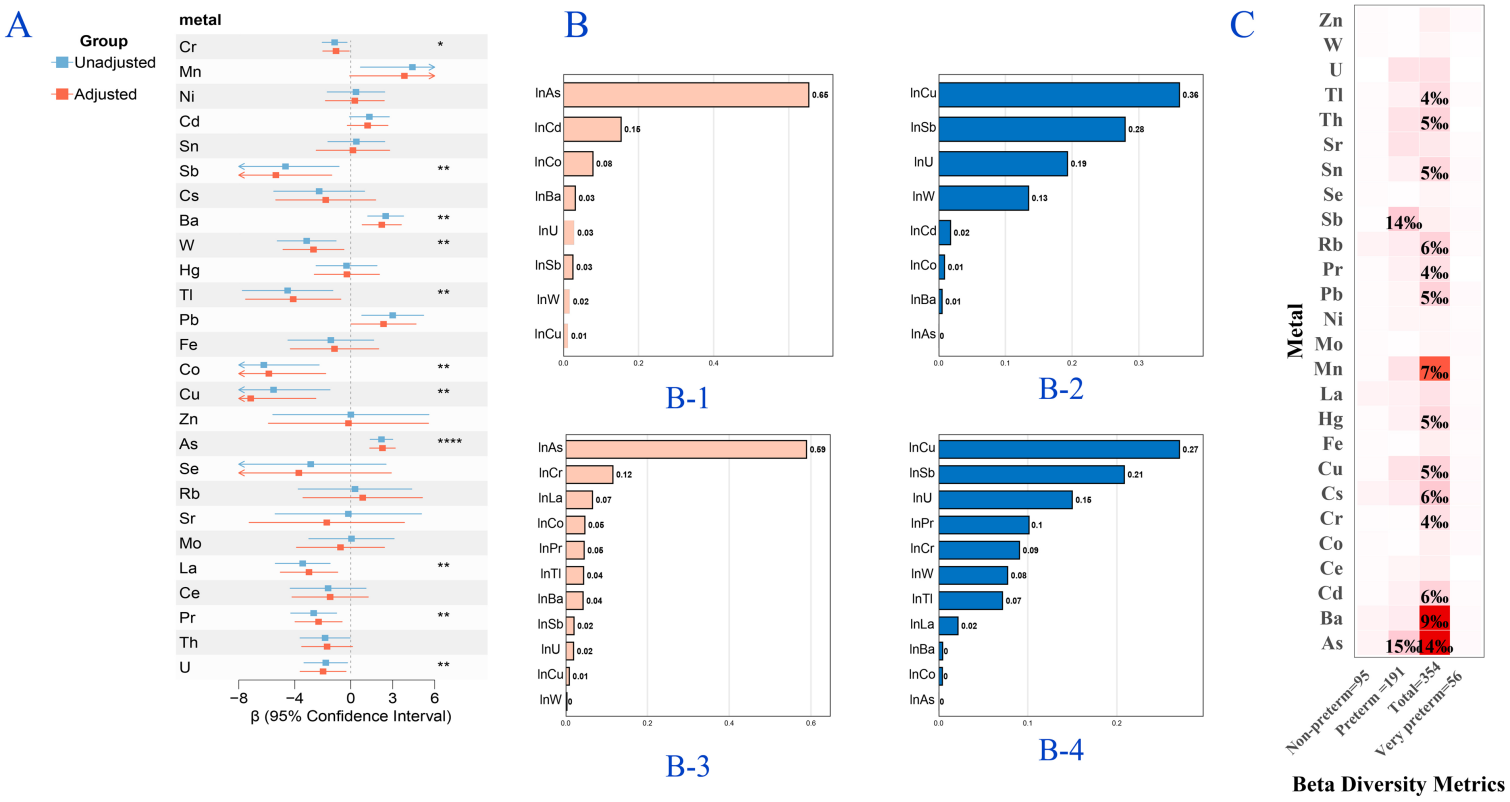
Associations between metal exposure and infant gut microbiota diversity metrics. All metal concentrations were ln-transformed. **(A)** Metal-specific associations with the Chao1 index (*q < 0.1, **q < 0.05, ***q < 0.01, ****q < 0.005). **(B)** Weighted quantile sum (WQS) regression results for metal mixtures and the Chao1 index: B-1/B-2 show elastic net-derived positive/negative metal weights, whereas B-3/B-4 show generalized linear model-derived weights. **(C)** Metal-beta diversity associations across preterm-stratified subgroups: (1) Total population, (2) Full term, (3) Preterm, and (4) Very preterm. Analyses are adjusted for sex, antibiotic exposure, breastfeeding history, delivery mode, birth weight, and age at sampling (additional preterm adjustment for the total population).

Further analysis (Figure 1A) indicated that Ba and As were positively associated with the Chao1 index, whereas Cr, Sb, W, Co, Cu, La, Pr, and U showed negative associations. These associations persisted in stratified analyses, demonstrating group-specific patterns: U remained significantly associated with the Chao1 index in full-term infants (*N* = 95), whereas Ba, Cu, and As showed significant associations in very preterm infants (*N* = 191). Notably, Sb and As maintained their associations with the Chao1 index in the very preterm (*N* = 56) (Supplementary Table S12).

**Figure 2:**
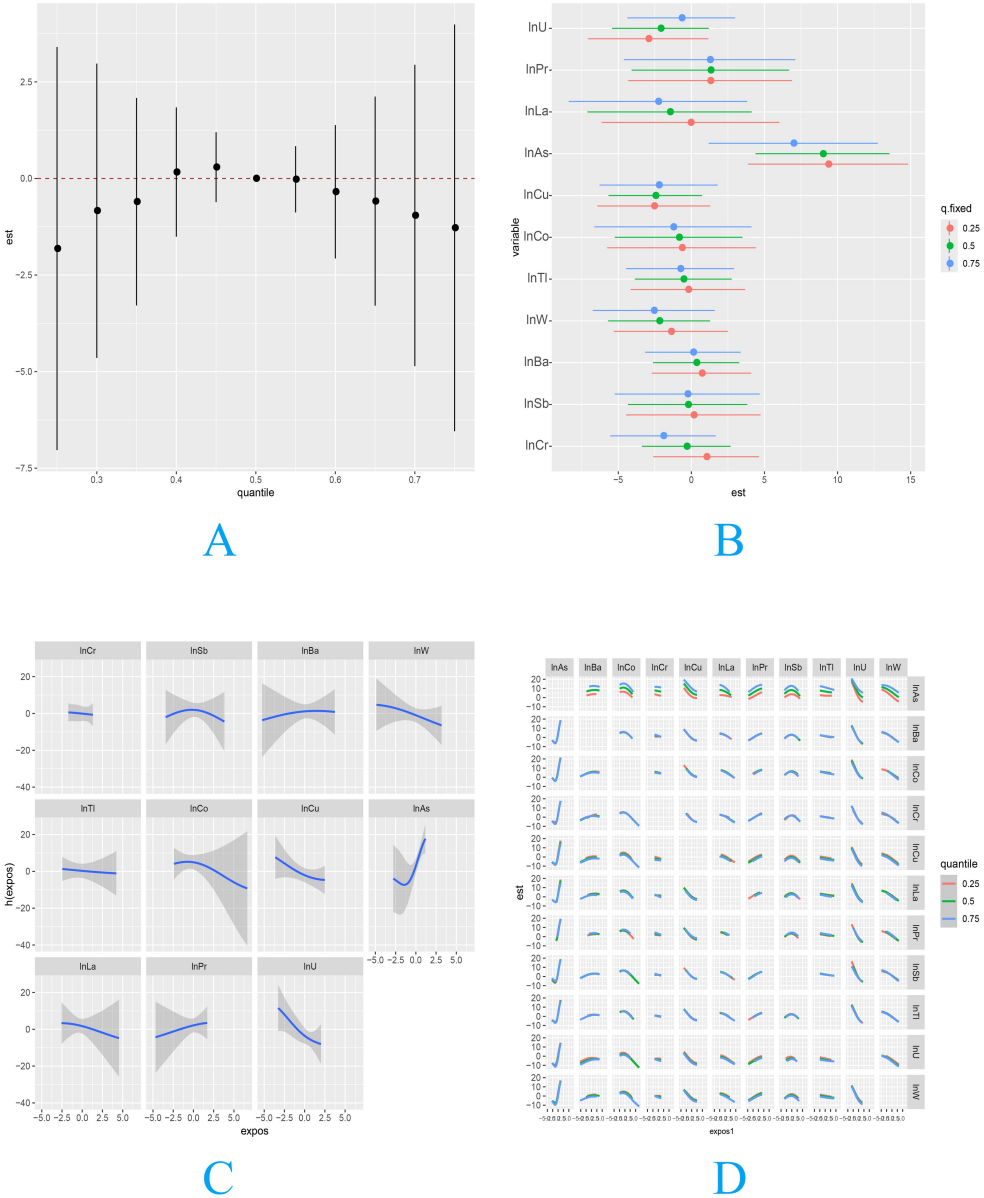
Associations of metal mixture exposure with the Chao1 index of infant gut microbiota. **(A)** Overall mixture effect on the Chao1 index (BKMR), **(B)** individual metal effects (conditional analysis), **(C)** dose–response relationships of key metals, **(D)** metal–metal interaction analysis.

The WQS regression (Figure 1B) revealed a statistically significant association between the metal mixture and Chao1 index, identifying As and Cu as components with the strongest positive and negative weights, respectively. This finding was further supported by BKMR analysis (Figure 2), which, while showing no overall mixture effect, consistently identified Sb, Cu, As, and U as key contributors (PIP > 0.7). Additional consistency was observed in both the elastic net and GLM-selected mixtures. All these associations were statistically significant (Supplementary Table S10). The combined multivariable analysis integrating BKMR and GLMs demonstrated significant interaction effects on the Chao1 index (Table 2). Six significant antagonistic interactions were detected, including Cr-W (*β* = −2.57, 95% CI: −4.49, −0.65), Cr-La (β = −3.82, 95% CI: −6.70, −0.94), Tl-As (β = −4.48, 95% CI: −7.57, −1.39), As-La (β = −4.31, 95%CI: −6.98, −1.64), As-Pr (β = −5.85, 95% CI: −9.23, −2.47), and La-Pr (β = −2.38, 95%CI: −4.26, −0.50) (q-interaction<0.1). Conversely, two synergistic interactions were identified: Sb-Pr (β = 2.17, 95% CI: 0.77, 3.58) and Sb-U (β = 2.14, 95% CI: 0.78, 3.50) (q-interaction<0.1).

**Table 2:** Metal–metal interactions (selected via single-metal GLMs) affecting the Chao index are shown as the interaction term *β* (95% CI).

Metals	Cr *β* (95%CI)	Sb β (95%CI)	Ba β (95%CI)	W β (95%CI)	Tl β (95%CI)	Co β (95%CI)	Cu β (95%CI)	As β (95%CI)	La β (95%CI)	Pr β (95%CI)	U β (95%CI)
Cr		−2.34(−4.41, −0.26)	−2.90(−5.58, −0.22)	−2.57(−4.49, −0.65) *	−1.73(−3.67, 0.21)	−1.24(−3.36, 0.87)	0.98(-0.78, 2.73)	−1.95(−7.31, 3.40)	−3.82(−6.70, −0.94) *	−2.56(−5.76, 0.65)	−1.12(−3.60, 1.36)
Sb			−0.29(−2.92, 2.33)	−0.84(−2.80, 1.13)	1.54(−0.05, 3.13)	−0.35(−2.09, 1.39)	−0.14(−2.14, 1.86)	−0.77 (−3.30, 1.75)	1.50(−0.32, 3.31)	2.17(0.77, 3.58) *	2.14(0.78, 3.50) *
Ba				−0.93(−3.02, 1.17)	1.88(-0.30, 4.07)	−0.25(−2.44, 1.94)	0.01(−1.80, 1.81)	0.46(−0.90, 1.81)	−1.09(−3.08, 0.89)	−0.68(−2.97, 1.61)	2.81(0.10, 5.53)
W					−1.46(−3.53, 0.61)	−2.16(−4.04, −0.28)	−0.08(−1.79, 1.63)	−2.00(−5.18, 1.17)	−1.05(−3.15, 1.04)	−1.20(−3.10, 0.70)	0.19(-1.82, 2.20)
Tl						−1.73(−3.39, −0.07)	0.35(−1.55, 2.26)	−4.48(−7.57, −1.39) *	−1.67(−3.66, 0.32)	−0.92(−2.78, 0.94)	2.17(0.30, 4.04)
Co							0.46(−1.44, 2.36)	−0.86(−2.59, 0.87)	−0.73(−2.45, 0.99)	0.26(−1.17, 1.69)	1.43(−0.13, 2.99)
Cu								−0.86(−2.77, 1.04)	0.18(−1.80, 2.17)	0.01(−2.16, 2.19)	−0.91(−3.02, 1.21)
As									−4.31(−6.98, −1.64) *	−5.85(−9.23, −2.47) *	0.24(−3.81, 4.30)
La										−2.38(−4.26, −0.50) *	−0.77(−2.76, 1.22)
Pr										−4.31 (−6.98, 1.64) *	

Models are adjusted for preterm birth, sex, antibiotic exposure, ever breastfed, delivery mode, birth weight, and age at sampling. Bold*: *q* < 0.1.

### 3.3 Beta diversity

Beta diversity, as quantified by weighted Bray–Curtis dissimilarity (Figure 1C), demonstrated a statistically significant association with As exposure that was robust to covariate adjustment (Supplementary Table S5). Nevertheless, the observed effect sizes were relatively small, accounting for approximately 1.4% of the variance (R^2^ ≈ 1.4%). This association was not maintained in subsequent stratified analyses, indicating a possible effect modification by the stratification variables.

### 3.4 Taxa (phylum and genus) associations

**Figure 3:**
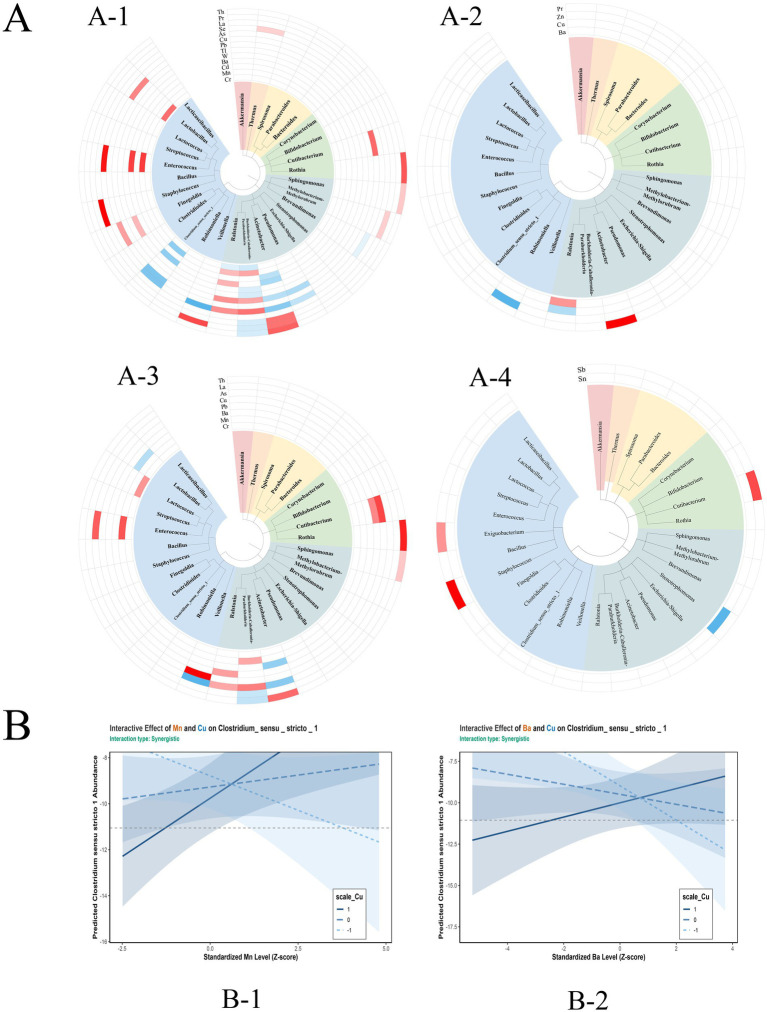
**(A)** Genus is statistically significantly associated either positively (blue) or negatively (red) with infant serum 26 metals. Group stratified by preterm birth. **(A-1)** Association of genus with metals in the general population (*N* = 342). **(A-2)** Association of genus with metals in the full-term group (*N* = 95). **(A-3)** The association of genus with metals in the preterm group (*N* = 191). **(A-4)** The association of genus with metals in the very-preterm group (*N* = 56). **(B)** Multivariate analysis of metal–metal interactions in association with the genus (*Clostridium_sensu_stricto_1*) (all *q* < 0.1). B-1: Manganese × Copper Interaction, B-2: Barium × Copper Interaction.

We examined 10 phyla and 30 genera that were present in more than 10% of the participants for associations with infant serum metal concentrations. Cr, Mn, Cd, Ba, W, Tl, Pb, Cu, As, Se, La, Pr, and Th were significantly associated with several genera after adjusting for covariates (Figure 3A; Supplementary Table S7).

In stratified analyses, distinct metal–microbiota associations were observed. In the full term (*N* = 95) (Figure 3A–2; Supplementary Table S15), Tl and U were significantly associated with the phylum (*Acidobacteriota*). Ba, Cu, Zn, and Pr were significantly associated with the abundances of several genera. In preterm infants (*N* = 191) (Figure 3A–3; Supplementary Table S16), Cs, Tl, and Se were significantly associated with the phylum (*Gemmatimonadota*). Cr, Mn, Ba, Pb, Cu, As, La, and Th were significantly associated with several genera. In very preterm infants (*N* = 56) (Figure 3A–4; Supplementary Table S17), Se was significantly associated with a phylum (*Verrucomicrobiota*). Sn and Sb were significantly associated with several genera.

BKMR analysis (Supplementary Figure S3; Supplementary Table S9) identified Mn as a key contributor to the abundance of *Burkholderia, Caballeronia,* and *Paraburkholderia* (PIP = 0.535). Exposure to a metal mixture (Cr, Mn, Ba, Pb, and As) was positively associated with *Ralstonia* abundance, with As showing the highest contribution (PIP = 0.886). Similarly, Cu demonstrated a substantial contribution to *Clostridium_sensu_stricto_1* abundance (PIP = 0.867). WQS analyses (Supplementary Table S10) revealed no significant associations between the metal mixture and the five genera examined. Multivariable analysis identified two synergistic interactions affecting *Clostridium_sensu_stricto_1* abundance (Figure 3B; Table 3): Mn-Cu (*β* = 0.797, 95% CI: 0.268–1.326) and Ba-Cu (β = 0.720, 95% CI: 0.220–1.220), both with q-interaction < 0.1.

**Table 3:** Metal–metal interactions (selected via single-metal MaAslin2) affecting the genus are shown as interaction term β (95% CI).

Genus	Metal:metal	β (95% CI)	*P*_value	FDR_q-Value
*Clostridium_sensu_stricto_1*	Mn:Ba	0.228(−0.238, 0.693)	0.338	0.406
	Mn:Cu	0.797(0.268, 1.326)	0.003	0.015*
	Mn:As	0.185 (−0.291, 0.661)	0.447	0.447
	Ba:Cu	0.720 (0.220, 1.220)	0.005	0.015*
	Ba:As	0.257 (−0.127, 0.641)	0.191	0.286
	Cu:As	0.422 (−0.121, 0.965)	0.128	0.256

Models are adjusted for preterm birth, sex, antibiotic exposure, ever breastfed, delivery mode, birth weight, and age at sampling. Bold*: *q* < 0.01.

## 4 Discussion

This study observes associations between early life metal exposure and infant gut microbiota colonization, which are reflected in distinct alterations in microbial abundance and alpha diversity, although with minimal effects on the overall community structure (beta diversity). These associations remained significant in both the preterm and very preterm subgroups. Analysis revealed six antagonistic metal–metal interactions (Cr-W, Cr-La, Tl-As, As-La, As-Pr, and La-Pr) alongside two synergistic interactions (Sb-Pr and Sb-U). In the mixture analyses, Mn was the primary contributor to *Burkholderia-Caballeronia-Paraburkholderia* abundance, whereas As showed the strongest association with *Ralstonia* abundance. Cu significantly influenced *Clostridium_sensu_stricto_1* abundance, with additional synergistic effects observed for the Mn-Cu and Ba-Cu combinations. These findings highlight the complex relationship between metal exposure and early gut microbiota development, underscoring the need for further mechanistic investigations.

Essential metal elements (Zoroddu et al., 2019; Jomova et al., 2022) are fundamental to a wide range of biological functions, with both deficiency and excess causing diverse pathological conditions. Among them, Mn, Fe, Co, Cu, Zn, Se, Mo, and Sr. are widely recognized as essential elements that perform vital biological roles (Rayman, 2000; Pajarillo et al., 2021; Huang X. Y. et al., 2022), including serving as components/cofactors of key enzymes, participating in electron transfer, and contributing to antioxidant reactions, among others.

This study employed Adonis analysis to assess the potential influence of Mn on microbial community structure, while MaAsLin2 analysis revealed that elevated serum Mn levels correlated with increased *Clostridium_sensu_stricto_1* abundance and decreased *Burkholderia-Caballeronia-Paraburkholderia, Enterococcus,* and *Ralstonia* abundances. These findings align with previous reports on the negative association of Mn with *Enterococcus* (Flores Ventura et al., 2024). However, contrasts exist regarding its inverse correlation with the Chao1 index (Flores Ventura et al., 2024) and its association with reduced *Verrucomicrobiota, Erysipelatoclostridiaceae, Eggerthellaceae, Akkermansiaceae,* and *Prevotellaceae* abundances (Shen et al., 2022). Fe exhibited no significant association with gut microbiota diversity or specific taxa, in contrast to earlier studies that demonstrated its influence on pediatric gut microbial composition (Laue et al., 2020). Co showed a negative association with the Chao1 index, aligning with findings that maternal Co-exposure reduces alpha diversity (Zhang et al., 2025) and is consistent with inverse Co-alpha diversity correlations in elderly populations (Zhang et al., 2023). Cu displayed a consistent negative association with the Chao1 index in BKMR and WQS analyses, along with inverse correlations with *Finegoldia, Cutibacterium, Lactobacillus,* and *Clostridium_sensu_stricto_1* abundances. Although no such associations have been reported in infant studies (Laue et al., 2020), similar reductions in *Lactobacillus* were observed in swine models (Meng et al., 2018; Brinck et al., 2023). Zn demonstrated no significant microbiota associations, although prior research has identified a synergistic negative relationship between Zn and As co-exposure and *Bifidobacterium abundance* in children (Laue et al., 2020). Se exhibited inverse correlations with *Staphylococcus* and *Thermus* abundances, a finding that was not replicated in infant populations. Mo showed no significant microbiota associations despite the reported links in the elderly and pregnant women (Zhang et al., 2023; Zhang et al., 2025).

In addition to essential metals, toxic metals (Cd, As, Pb, Hg, Cr, Tl, and Sb) present health risks because of their adverse biological effects (Bist and Choudhary, 2022; Wysocki et al., 2023; Peng et al., 2024; Zhao et al., 2025). In our study, Cd was significantly associated with increased *Acinetobacter* abundance, a finding not previously reported in humans but supported by murine models (Li et al., 2019; He et al., 2020). It exhibited strong positive associations with the Chao1 index and was the most influential element in the BKMR and WQS analyses, while also affecting beta diversity. It consistently reduced *Ralstonia, Enterococcus,* and *Burkholderia-Caballeronia-Paraburkholderia* abundances, but increased *Acinetobacter, Veillonella, Clostridium_sensu_stricto_1*, *Pseudomonas,* and *Brevundimonas.* Critically, both analytical approaches identified arsenic as a key driver of mixture effects on *Ralstonia,* with most associations persisting in preterm infants. These results align with prior reports of As reducing *Enterococcus* in infants (Laue et al., 2020) and altering gut microbiota in mice (Chi et al., 2017). Pb was associated with lower *Ralstonia,* and *Burkholderia-Caballeronia-Paraburkholderia,* but higher *Pseudomonas* and *Acinetobacter*, diverging from infant studies (Eggers et al., 2019; Sitarik et al., 2020; Zeng et al., 2022). Cr was negatively correlated with the Chao1 index and reduced *Lactobacillus* while increasing *Burkholderia-Caballeronia-Paraburkholderia*, and *Ralstonia*, although these findings were not replicated in infants (Xiang et al., 2024) or mice (Yan et al., 2023). Higher Tl levels increase *Burkholderia-Caballeronia-Paraburkholderia*, a trend absent in infant studies (Xiang et al., 2024).

Notably, the potential impacts of several rare elements (Ba, W), rare earth elements (La, Pr), and radioactive metals (Th, U) on the gut microbiota remain poorly characterized in existing literature. Ba was associated with a reduced Chao1 index and a lower abundance of *Enterococcus* and *Ralstonia,* while increasing *Clostridium_sensu_stricto_1* and *Acinetobacter,* unlike infant data (Xiang et al., 2024). W persistently lowered the Chao1 index in preterm infants and reduced the abundance of *Finegoldia*, *Burkholderia-Caballeronia-Paraburkholderia*, consistent with its antibacterial effects (Qin et al., 2025). La was associated with reductions in *Acinetobacter* and increases in *Methylobacterium and Methylorubrum,* but increased *Burkholderia-Caballeronia-Paraburkholderia*. Similarly, Pr was associated with an elevated abundance of *Burkholderia-Caballeronia-Paraburkholderia* and reductions in *Acinetobacter* and *Veillonella*. This was associated with decreased *Acinetobacter, Rothia,* and *Sphingomonas* but an increase in *Burkholderia-Caballeronia-Paraburkholderia*. Our study reveals more infant microbiota associations with these rare metals than previously reported, addressing a critical research gap. U showed a negative association with alpha diversity, expanding evidence on early life metal–microbiota interactions.

Metal exposure showed differential associations with gut microbiota across infant groups. In preterm infants, Ba was negatively correlated with the Chao1 index, whereas Cs and Sb showed significant associations in very preterm infants. Notably, W and Cu were associated with the Chao1 index in both the preterm and very preterm groups, indicating the heightened sensitivity of premature infant microbiota to metalloid/metal exposure. Full-term infants exhibited distinct patterns, with Tl and U associated with a single phylum versus Ba, Cu, Zn, and Pr, correlating with multiple genera. Preterm infants demonstrated phylum-level associations with Cs, Tl, and Se, and genus-level links with Cr, Mn, Ba, Pb, Cu, As, La, and Th. Very preterm infants showed phylum-level associations with Se and genus-level associations with Sn and Sb. These findings may reflect known associations between W, Ba, Cu, and As and preterm birth and subsequent elevated metal exposure (Jiang et al., 2018; Huang et al., 2021; Karakis et al., 2021; Liu C. et al., 2022). Because the gut ecosystem in preterm infants is particularly vulnerable (Cuna et al., 2021), these findings suggest that their microbiota may be more responsive to metalloid/metal effects. Further investigation is needed to elucidate the complex relationships between metal exposure, preterm birth, and gut microbiota development.

BKMR analyses identified Mn as a key contributor to *Burkholderia-Caballeronia-Paraburkholderia*, which was positively correlated with sodium taurocholate (STCA) and sodium taurodeoxycholate (STDCA) and negatively correlated with bile salt hydrolase (BSH) and hydroxysteroid dehydrogenase (HSDH) content (Lei et al., 2021). It has a high contribution to *Ralstonia,* which has been shown to cause infections that are sometimes serious, such as osteomyelitis and meningitis, in hospital settings (Ryan and Adley, 2014). Cu had a high contribution to *Clostridium_sensu_stricto_1*, which might be the pivotal pathogenic bacteria of polycystic ovarian syndrome with insulin resistance (PCOS-IR) (Zhao et al., 2022). Collectively, these metal-sensitive genera may represent key microbial targets in gut ecosystems. However, the current understanding remains limited and warrants further mechanistic validation through large-scale cohort studies and experimental investigations.

Our study revealed more essential/toxic metal and infant gut microbiota associations than previously reported, with novel documentation of metal–metal interactions—six antagonistic (Cr-W, Cr-La, Tl-As, As-La, As-Pr, and La-Pr) and two synergistic (Sb-Pr and Sb-U) pairs influencing microbial composition. The Mn-Cu and Ba-Cu combinations demonstrated synergistic effects on *Clostridium_sensu_stricto_1* abundance. Notably, prior studies have reported that Mn-Cu interactions affect WBC count (Huang C. H. et al., 2022), spontaneous preterm birth risk (Issah et al., 2024), and grip strength (Liang et al., 2024). Their combined effects on gut microbiota remain undocumented in the existing literature. These interactions warrant further investigation and validation to elucidate their underlying mechanisms and better protect children’s health.

Microbial function prediction analysis further revealed that the associations of key metals (Cu and As) with the gut microbiota may involve several core metabolic and cellular pathways (Supplementary Figure S4), including Propanoate metabolism, Glycosaminoglycan degradation, Pantothenate and CoA biosynthesis, Peroxisome, One carbon pool by folate, Glycine, serine and threonine metabolism, Lysosome, Glutathione metabolism, and Biosynthesis of amino acids. These pathways suggest that metal exposure might influence microbiota-host interactions by interfering with core biological processes such as short-chain fatty acid production, cellular energy metabolism, the antioxidant defense system (glutathione), and organelle function. This aligns with previous research indicating that metal exposure can affect the production of metabolites like short-chain fatty acids and bile acids, vitamin metabolism, as well as ATP synthesis and redox homeostasis (Bist and Choudhary, 2022; Nehzomi and Shirani, 2024). Furthermore, alterations in Glycosaminoglycan degradation and Lysosome pathways also imply that metals may modulate inflammatory responses by affecting innate immune signaling pathways such as proteoglycan/TLR2 and LPS/TLR4 (Teffera et al., 2024).

Building upon these findings regarding health risks, future strategies could explore gut-targeted bioremediation for mitigating the effects of metal exposure. Previous studies have indicated that the gut microbiota and specific probiotics represent validated, effective, and economical therapeutic strategies for alleviating heavy metal toxicity in humans (Arun et al., 2021). Therefore, dietary supplementation with specific probiotic species to modulate the gut microbiota could emerge as a moderate, cost-effective, and efficient auxiliary strategy for mitigating heavy metal pollutants in the human body, offering a promising direction for future public health interventions.

This pioneering study investigates 26 serum metals and their associations with gut microbiota in hospitalized infants, encompassing both preterm and other clinical conditions. We demonstrate significant relationships between metal exposure and gut health in this vulnerable population, revealing novel associations between rare earth elements (REEs) and radionuclides. Importantly, we identified the metal interaction effects that influenced both the Chao1 index and *Clostridium_sensu_stricto_1* abundance. Premature infants show a particular susceptibility in alpha diversity and specific taxa to metalloid/metal exposure, highlighting the fragility of their developing gut ecosystems. However, this study had several limitations. First, because of its cross-sectional design, it is difficult to establish a causal relationship between metal exposure and the gut microbiome. We will conduct causal arguments through further experiments and cohort studies. Second, the single-center hospital-based design may have introduced Berkson’s bias, where hospitalization is related to both metal exposure and microbiome composition, potentially affecting the accuracy of the association estimates and limiting generalizability. Future validation in community-based cohorts and the use of methods such as inverse probability weighting to address this bias are therefore necessary. Finally, despite the inclusion of many covariates for adjustment, the included infants did not consider the impact of underlying diseases and environmental exposure during the prenatal and postnatal periods.

## 5 Conclusion

This study identifies arsenic and copper as the metals most strongly associated with gut microbial alpha diversity, whereas copper, arsenic, and manganese are significantly associated with the abundance of specific microbial taxa. By providing novel epidemiological evidence on metal-gut microbiota interactions in vulnerable infants, our findings suggest that pollutant exposure may critically disrupt microbiome development, with potential long-term health implications. Further mechanistic and population-based research is imperative to validate these associations, elucidate the underlying biological pathways, and assess their clinical significance for child health outcomes.

## Data Availability

The datasets presented in this study can be found in online repositories. The names of the repository/repositories and accession number(s) can be found below: https://www.ncbi.nlm.nih.gov/sra, PRJNA814846.
