# Supplementary material for: Association between infants’ serum levels of 26 metals and gut microbiota: a hospital-based cross-sectional study in China
Source: Front Microbiol. 2025 Dec 11;16:1669475. doi: 10.3389/fmicb.2025.1669475 (PMC12740241; doi:10.3389/fmicb.2025.1669475)
Supplement: Supplementary file 1 [file Data_Sheet_1.docx]

**Supplemental Material**

**Association between infants’serum 26 metals and gut microbiota in China: a hospital-based cross-sectional study**

Xing Yan1†, Jun Qiu12†，Ruiwen Huang3, Xiaoming Peng3, Shi-ting Xiang2, Kunyan Zhao4, Yunlong Peng5, Yan Zhuang3, Ye Ma3, Mingyang Wu1*, Fei Yang14*

**1. Details of the detection method**

**1.1 Metal Measurement**

26 metals/metalloids were measured, including chromium (Cr), manganese (Mn), nickel (Ni), cadmium (Cd), tin (Sn), antimony (Sb), cesium (Cs), barium (Ba), tungsten (W), mercury (Hg), thallium (Tl), lead (Pb), iron (Fe), cobalt (Co), copper (Cu), zinc (Zn), arsenic (As), selenium (Se), rubidium (Rb), strontium (Sr), molybdenum (Mo), lanthanum (La), cerium (Ce), praseodymium (Pr), thorium (Th), and uranium (U). Their limits of detection (LOD) are provided in the supplementary materials (Table S2). The concentrations of metals in serum were measured using inductively coupled plasma mass spectrometry (ICP-MS; PerkinElmer Nex ION 1000G, USA). To prevent contamination, all samples were diluted at an ultra-clean workbench, and metal contact during the analysis was strictly avoided. A blank control was used during instrument analysis to subtract the background value, ensuring accuracy. Quality control of the serum metal determinations was achieved through three repeated measurements, calculation of the recovery rate of the labeled mixed serum sample, and comparison with the reference range provided by Clin Chek® Controls (No. 8882).

**Table S1 Association of alpha diversity with individual covariate (**Generalized linear regression)

| Diversity Matrix | Covariates | Estimate(β) | p value |
| --- | --- | --- | --- |
| Shannon | Sex | 0.932 | 0.281 |
|  | **Antibiotic** | **1.234** | **0.004** |
|  | Ever Breastfed | 1.006 | 0.922 |
|  | Delivery Mode | 0.89 | 0.067 |
|  | **Birth Weight** | **-8.73E-05** | **0.031** |
|  | **Gestational Age** | **-0.02** | **0.034** |
|  | Sample Age | 0.002 | 0.203 |
| Simpson | Sex | 0.987 | 0.599 |
|  | **Antibiotic** | **1.075** | **0.007** |
|  | Ever Breastfed | 0.995 | 0.824 |
|  | Delivery Mode | 0.957 | 0.148 |
|  | Birth Weight | -2.92E-05 | 0.053 |
|  | **Gestational Age** | **-0.007** | **0.046** |
|  | Sample Age | 0.001 | 0.214 |
| Chao | Sex | 1.092 | 0.963 |
|  | Antibiotic | 0.052 | 0.163 |
|  | **Ever Breastfed** | **88.432** | **0.016** |
|  | Delivery Mode | 0.028 | 0.054 |
|  | Birth Weight | 0.00 | 0.826 |
|  | Gestational Age | -0.102 | 0.714 |
|  | Sample Age | -0.04 | 0.324 |
| Pielou | Sex | 0.989 | 0.362 |
|  | **Antibiotic** | **1.048** | **0.000304** |
|  | Ever Breastfed | 0.994 | 0.591 |
|  | Delivery Mode | 0.983 | 0.135 |
|  | **Birth Weight** | **-1.59E-05** | **0.028** |
|  | **Gestational Age** | **-0.004** | **0.037** |
|  | Sample Age | 0.00 | 0.102 |

**Table S2 Distribution of 26 metals levels in serum (****μg/L)**

| Metals | ≥LOD% | LOD | Geometric mean | Percentile | | | | |
| --- | --- | --- | --- | --- | --- | --- | --- | --- |
|  |  |  |  | P5 | P25 | P50 | P75 | P95 |
| Cr | 75.73 | 0.039044 | 2.75 | 0.00 | 0.76 | 2.36 | 3.36 | 6.26 |
| Mn | 100.00 | 0.001728 | 2.32 | 1.12 | 1.67 | 2.23 | 3.07 | 5.36 |
| Ni | 99.70 | 0.001108 | 1.61 | 0.56 | 1.02 | 1.50 | 2.34 | 6.65 |
| Cd | 94.15 | 0.001361 | 0.09 | 0.00 | 0.05 | 0.09 | 0.13 | 0.34 |
| Sn | 100.00 | 0.002570 | 0.65 | 0.20 | 0.34 | 0.58 | 1.09 | 3.33 |
| Sb | 100.00 | 0.000972 | 6.84 | 3.10 | 5.25 | 6.88 | 9.14 | 14.50 |
| Cs | 100.00 | 0.000252 | 0.87 | 0.31 | 0.64 | 0.90 | 1.25 | 2.01 |
| Ba | 98.83 | 0.002163 | 2.51 | 0.37 | 1.28 | 2.26 | 4.61 | 17.51 |
| W | 99.42 | 0.001906 | 0.09 | 0.03 | 0.05 | 0.08 | 0.15 | 0.31 |
| Hg | 99.70 | 0.001090 | 0.30 | 0.08 | 0.18 | 0.27 | 0.49 | 1.10 |
| Tl | 100.00 | 0.000197 | 0.02 | 0.01 | 0.01 | 0.02 | 0.03 | 0.05 |
| Pb | 100.00 | 0.002599 | 0.85 | 0.23 | 0.49 | 0.83 | 1.43 | 3.18 |
| Fe | 100.00 | 0.121541 | 1691.98 | 761.10 | 1132.66 | 1514.24 | 2354.71 | 5009.13 |
| Co | 100.00 | 0.000292 | 0.22 | 0.12 | 0.16 | 0.21 | 0.29 | 0.42 |
| Cu | 100.00 | 0.002028 | 455.97 | 211.33 | 341.77 | 454.26 | 616.38 | 937.02 |
| Zn | 100.00 | 0.117646 | 598.43 | 372.66 | 487.07 | 582.31 | 707.37 | 974.89 |
| As | 90.35 | 0.001578 | 0.90 | 0.00 | 0.41 | 0.90 | 1.67 | 3.11 |
| Se | 100.00 | 0.073494 | 48.37 | 25.92 | 40.72 | 50.73 | 60.65 | 76.19 |
| Rb | 100.00 | 0.000756 | 248.33 | 125.84 | 194.62 | 246.28 | 319.46 | 490.33 |
| Sr | 100.00 | 0.004735 | 23.81 | 13.48 | 19.11 | 23.95 | 29.34 | 41.70 |
| Mo | 100.00 | 0.001619 | 1.26 | 0.46 | 0.85 | 1.25 | 1.88 | 3.25 |
| La | 100.00 | 0.000355 | 0.12 | 0.03 | 0.06 | 0.10 | 0.21 | 0.58 |
| Ce | 100.00 | 0.000003 | 0.18 | 0.06 | 0.12 | 0.18 | 0.27 | 0.53 |
| Pr | 99.12 | 0.000218 | 0.03 | 0.0048 | 0.01 | 0.02 | 0.05 | 0.13 |
| Th | 99.70 | 0.001167 | 0.22 | 0.03 | 0.12 | 0.23 | 0.44 | 0.93 |
| U | 100.00 | 0.000146 | 0.52 | 0.03 | 0.46 | 0.75 | 1.03 | 1.64 |

**Table S3 Unadjusted associations of ln-transformed metals with alpha diversity.** The effect size estimate expressed in the coef column represents the changes in species relative abundance per unit increase in blood metals. ci_low represents 95% confidence interval lower bounds, ci_high represents 95% confidence interval upper bounds. *P* value (pval column) calculated from Generalized linear regression. False discovery rate (FDR) adjusted q values are in the last column (FDR qval). q* < 0.1 was considered statistically significant.

| Alpha_ diversity | Metals | coef | t_value | ci_low | ci_high | pval | FDR_qval |
| --- | --- | --- | --- | --- | --- | --- | --- |
| Shannon | Cr | -0.011 | -0.709 | -0.042 | 0.020 | 0.479 | 0.778375 |
|  | Mn | 0.026 | 0.393 | -0.103 | 0.154 | 0.694 | 0.8934545 |
|  | Ni | -0.015 | -0.417 | -0.086 | 0.056 | 0.677 | 0.8934545 |
|  | Cd | 0.001 | 0.034 | -0.048 | 0.050 | 0.973 | 0.973 |
|  | Sn | -0.051 | -1.426 | -0.120 | 0.019 | 0.155 | 0.4368 |
|  | Sb | -0.093 | -1.380 | -0.225 | 0.039 | 0.168 | 0.4368 |
|  | Cs | -0.135 | -2.387 | -0.246 | -0.024 | **0.018** | 0.2340000 |
|  | Ba | 0.017 | 0.737 | -0.028 | 0.062 | 0.462 | 0.778375 |
|  | W | -0.018 | -0.482 | -0.091 | 0.055 | 0.630 | 0.8934545 |
|  | Hg | -0.061 | -1.610 | -0.136 | 0.013 | 0.108 | 0.4368 |
|  | Tl | -0.154 | -2.713 | -0.265 | -0.043 | **0.007** | 0.1820000 |
|  | Pb | 0.002 | 0.050 | -0.075 | 0.079 | 0.960 | 0.973 |
|  | Fe | -0.075 | -1.394 | -0.180 | 0.030 | 0.164 | 0.4368 |
|  | Co | -0.135 | -1.930 | -0.272 | 0.002 | 0.054 | 0.4368 |
|  | Cu | -0.116 | -1.637 | -0.256 | 0.023 | 0.103 | 0.4368 |
|  | Zn | -0.122 | -1.247 | -0.313 | 0.069 | 0.213 | 0.5034545 |
|  | As | 0.015 | 1.035 | -0.014 | 0.044 | 0.301 | 0.602 |
|  | Se | 0.024 | 0.252 | -0.161 | 0.209 | 0.801 | 0.9054783 |
|  | Rb | -0.100 | -1.413 | -0.239 | 0.039 | 0.159 | 0.4368 |
|  | Sr | 0.006 | 0.069 | -0.173 | 0.186 | 0.945 | 0.973 |
|  | Mo | 0.076 | 1.424 | -0.029 | 0.180 | 0.156 | 0.4368 |
|  | La | -0.011 | -0.322 | -0.080 | 0.057 | 0.748 | 0.8934545 |
|  | Ce | -0.016 | -0.344 | -0.110 | 0.077 | 0.731 | 0.8934545 |
|  | Pr | -0.033 | -1.137 | -0.090 | 0.024 | 0.256 | 0.5546667 |
|  | Th | -0.010 | -0.311 | -0.072 | 0.052 | 0.756 | 0.8934545 |
|  | U | -0.022 | -0.784 | -0.075 | 0.032 | 0.434 | 0.778375 |
| Simpson | Cr | -0.001 | -0.177 | -0.013 | 0.010 | 0.860 | 0.983 |
|  | Mn | 0.014 | 0.562 | -0.034 | 0.062 | 0.575 | 0.9176471 |
|  | Ni | -0.010 | -0.741 | -0.036 | 0.016 | 0.459 | 0.832 |
|  | Cd | 0.001 | 0.101 | -0.017 | 0.019 | 0.919 | 0.983 |
|  | Sn | -0.022 | -1.628 | -0.047 | 0.004 | 0.104 | 0.507 |
|  | Sb | -0.023 | -0.902 | -0.072 | 0.027 | 0.368 | 0.832 |
|  | Cs | -0.052 | -2.486 | -0.094 | -0.011 | **0.013** | 0.169 |
|  | Ba | 0.006 | 0.730 | -0.011 | 0.023 | 0.466 | 0.832 |
|  | W | -0.006 | -0.437 | -0.033 | 0.021 | 0.663 | 0.9576667 |
|  | Hg | -0.026 | -1.838 | -0.054 | 0.002 | 0.067 | 0.507 |
|  | Tl | -0.062 | -2.927 | -0.103 | -0.020 | **0.004** | 0.104 |
|  | Pb | -0.001 | -0.094 | -0.030 | 0.027 | 0.925 | 0.983 |
|  | Fe | -0.014 | -0.706 | -0.053 | 0.025 | 0.480 | 0.832 |
|  | Co | -0.041 | -1.572 | -0.092 | 0.010 | 0.117 | 0.507 |
|  | Cu | -0.026 | -0.990 | -0.078 | 0.026 | 0.323 | 0.832 |
|  | Zn | -0.043 | -1.181 | -0.114 | 0.028 | 0.239 | 0.77675 |
|  | As | 0.003 | 0.524 | -0.008 | 0.014 | 0.600 | 0.9176471 |
|  | Se | -0.001 | -0.022 | -0.070 | 0.068 | 0.983 | 0.983 |
|  | Rb | -0.045 | -1.700 | -0.097 | 0.007 | 0.090 | 0.507 |
|  | Sr | -0.003 | -0.087 | -0.070 | 0.064 | 0.931 | 0.983 |
|  | Mo | 0.028 | 1.389 | -0.011 | 0.066 | 0.166 | 0.6165714 |
|  | La | 0.001 | 0.106 | -0.024 | 0.027 | 0.916 | 0.983 |
|  | Ce | -0.001 | -0.048 | -0.036 | 0.034 | 0.962 | 0.983 |
|  | Pr | -0.009 | -0.793 | -0.030 | 0.013 | 0.429 | 0.832 |
|  | Th | -0.002 | -0.185 | -0.025 | 0.021 | 0.853 | 0.983 |
|  | U | -0.008 | -0.781 | -0.028 | 0.012 | 0.435 | 0.832 |
| Chao | Cr | -1.148 | -2.516 | -2.042 | -0.254 | **0.012** | **0.0312*** |
|  | Mn | 4.406 | 2.325 | 0.691 | 8.122 | **0.021** | **0.0455*** |
|  | Ni | 0.376 | 0.358 | -1.684 | 2.437 | 0.721 | 0.892666667 |
|  | Cd | 1.334 | 1.830 | -0.094 | 2.762 | 0.068 | 0.117866667 |
|  | Sn | 0.401 | 0.386 | -1.636 | 2.439 | 0.700 | 0.892666667 |
|  | Sb | -4.660 | -2.384 | -8.491 | -0.829 | **0.018** | **0.042545455*** |
|  | Cs | -2.254 | -1.359 | -5.505 | 0.998 | 0.175 | 0.284375 |
|  | Ba | 2.496 | 3.804 | 1.210 | 3.782 | **0.00017** | **0.00221*** |
|  | W | -3.147 | -2.926 | -5.255 | -1.039 | **0.004** | **0.017333333*** |
|  | Hg | -0.298 | -0.268 | -2.479 | 1.883 | 0.789 | 0.932454545 |
|  | Tl | -4.509 | -2.728 | -7.748 | -1.270 | **0.007** | **0.023111111*** |
|  | Pb | 3.000 | 2.656 | 0.786 | 5.214 | **0.008** | **0.023111111*** |
|  | Fe | -1.422 | -0.908 | -4.491 | 1.648 | 0.365 | 0.499473684 |
|  | Co | -6.209 | -3.075 | -10.165 | -2.252 | **0.002** | **0.0104*** |
|  | Cu | -5.514 | -2.677 | -9.552 | -1.476 | **0.008** | **0.023111111*** |
|  | Zn | 0.011 | 0.004 | -5.571 | 5.593 | 0.997 | 0.997 |
|  | As | 2.197 | 5.307 | 1.386 | 3.009 | **0.0000002** | **0.0000052*** |
|  | Se | -2.859 | -1.040 | -8.246 | 2.529 | 0.299 | 0.431888889 |
|  | Rb | 0.307 | 0.148 | -3.756 | 4.370 | 0.882 | 0.997 |
|  | Sr | -0.170 | -0.064 | -5.405 | 5.064 | 0.949 | 0.997 |
|  | Mo | 0.054 | 0.035 | -2.997 | 3.106 | 0.972 | 0.997 |
|  | La | -3.430 | -3.427 | -5.392 | -1.468 | **0.0007** | **0.006066667*** |
|  | Ce | -1.615 | -1.165 | -4.332 | 1.102 | 0.245 | 0.374705882 |
|  | Pr | -2.647 | -3.153 | -4.292 | -1.001 | **0.002** | **0.0104*** |
|  | Th | -1.831 | -1.999 | -3.625 | -0.036 | **0.046** | **0.085428571*** |
|  | U | -1.781 | -2.235 | -3.343 | -0.220 | **0.026** | **0.052*** |
| Pielou | Cr | 0.000 | 0.144 | -0.005 | 0.006 | 0.886 | 0.97825 |
|  | Mn | -0.001 | -0.122 | -0.024 | 0.022 | 0.903 | 0.97825 |
|  | Ni | -0.004 | -0.617 | -0.017 | 0.009 | 0.538 | 0.9058947 |
|  | Cd | -0.003 | -0.563 | -0.011 | 0.006 | 0.574 | 0.9058947 |
|  | Sn | -0.010 | -1.554 | -0.022 | 0.003 | 0.121 | 0.5243333 |
|  | Sb | -0.009 | -0.730 | -0.033 | 0.015 | 0.466 | 0.9058947 |
|  | Cs | -0.021 | -2.027 | -0.041 | -0.001 | **0.043** | 0.5243333 |
|  | Ba | -0.002 | -0.367 | -0.010 | 0.007 | 0.714 | 0.9282 |
|  | W | 0.003 | 0.437 | -0.010 | 0.016 | 0.662 | 0.9058947 |
|  | Hg | -0.011 | -1.586 | -0.024 | 0.003 | 0.114 | 0.5243333 |
|  | Tl | -0.022 | -2.131 | -0.042 | -0.002 | **0.034** | 0.5243333 |
|  | Pb | -0.005 | -0.747 | -0.019 | 0.009 | 0.455 | 0.9058947 |
|  | Fe | -0.009 | -0.943 | -0.028 | 0.010 | 0.347 | 0.8816364 |
|  | Co | -0.012 | -0.939 | -0.036 | 0.013 | 0.348 | 0.8816364 |
|  | Cu | -0.012 | -0.954 | -0.037 | 0.013 | 0.341 | 0.8816364 |
|  | Zn | -0.024 | -1.351 | -0.058 | 0.011 | 0.178 | 0.6611429 |
|  | As | -0.001 | -0.509 | -0.007 | 0.004 | 0.611 | 0.9058947 |
|  | Se | 0.010 | 0.572 | -0.024 | 0.043 | 0.568 | 0.9058947 |
|  | Rb | -0.021 | -1.677 | -0.046 | 0.004 | 0.094 | 0.5243333 |
|  | Sr | 0.001 | 0.054 | -0.031 | 0.033 | 0.957 | 0.994 |
|  | Mo | 0.015 | 1.616 | -0.003 | 0.034 | 0.107 | 0.5243333 |
|  | La | 0.006 | 0.893 | -0.007 | 0.018 | 0.373 | 0.8816364 |
|  | Ce | 0.002 | 0.276 | -0.014 | 0.019 | 0.783 | 0.9694286 |
|  | Pr | -0.001 | -0.135 | -0.011 | 0.010 | 0.893 | 0.97825 |
|  | Th | 0.003 | 0.486 | -0.008 | 0.014 | 0.628 | 0.9058947 |
|  | U | 0.000 | -0.007 | -0.010 | 0.010 | 0.994 | 0.994 |

**Table S4 Adjusted associations of ln-transformed metals with alpha diversity.** The effect size estimate expressed in the coef column represents the changes in species relative abundance per unit increase in blood metals. ci_low represents 95% confidence interval lower bounds, ci_high represents 95% confidence interval upper bounds. *P* value (pval column) calculated from Generalized linear regression. False discovery rate (FDR) adjusted q values are in the last column (FDR qval). q* < 0.1 was considered statistically significant. Models were adjusted for Preterm, Sex, Antibiotic, Ever Breastfed, Delivery Mode, Birth Weight and Sample Age.

| Alpha_ diversity | Metals | coef | t_value | ci_low | ci_high | pval | FDR_qval |
| --- | --- | --- | --- | --- | --- | --- | --- |
| Shannon | Cr | -0.017 | -1.018 | -0.049 | 0.015 | 0.309 | 0.8034 |
|  | Mn | 0.092 | 1.374 | -0.039 | 0.224 | 0.170 | 0.5525 |
|  | Ni | 0.007 | 0.207 | -0.063 | 0.078 | 0.836 | 0.86944 |
|  | Cd | 0.009 | 0.364 | -0.040 | 0.058 | 0.716 | 0.86944 |
|  | Sn | -0.012 | -0.267 | -0.100 | 0.076 | 0.789 | 0.86944 |
|  | Sb | -0.045 | -0.651 | -0.180 | 0.090 | 0.515 | 0.86944 |
|  | Cs | -0.113 | -1.853 | -0.233 | 0.007 | 0.065 | 0.416 |
|  | Ba | 0.035 | 1.455 | -0.012 | 0.083 | 0.147 | 0.5525 |
|  | W | -0.016 | -0.429 | -0.090 | 0.058 | 0.668 | 0.86944 |
|  | Hg | -0.009 | -0.237 | -0.088 | 0.069 | 0.813 | 0.86944 |
|  | Tl | -0.145 | -2.486 | -0.259 | -0.031 | **0.013** | 0.1386667 |
|  | Pb | 0.021 | 0.511 | -0.058 | 0.099 | 0.609 | 0.86944 |
|  | Fe | -0.016 | -0.290 | -0.122 | 0.090 | 0.772 | 0.86944 |
|  | Co | -0.085 | -1.210 | -0.223 | 0.053 | 0.227 | 0.6557778 |
|  | Cu | -0.250 | -3.146 | -0.407 | -0.094 | **0.002** | **0.052*** |
|  | Zn | -0.081 | -0.827 | -0.273 | 0.111 | 0.409 | 0.8115714 |
|  | As | 0.039 | 2.421 | 0.007 | 0.071 | **0.016** | 0.1386667 |
|  | Se | 0.011 | 0.095 | -0.211 | 0.233 | 0.924 | 0.924 |
|  | Rb | -0.018 | -0.248 | -0.161 | 0.125 | 0.805 | 0.86944 |
|  | Sr | -0.057 | -0.599 | -0.243 | 0.129 | 0.549 | 0.86944 |
|  | Mo | 0.094 | 1.756 | -0.011 | 0.199 | 0.080 | 0.416 |
|  | La | -0.028 | -0.778 | -0.097 | 0.042 | 0.437 | 0.8115714 |
|  | Ce | -0.014 | -0.300 | -0.106 | 0.078 | 0.764 | 0.86944 |
|  | Pr | -0.040 | -1.375 | -0.097 | 0.017 | 0.170 | 0.5525 |
|  | Th | -0.025 | -0.783 | -0.086 | 0.037 | 0.434 | 0.8115714 |
|  | U | -0.026 | -0.928 | -0.082 | 0.029 | 0.354 | 0.8115714 |
| Simpson | Cr | -0.003 | -0.414 | -0.015 | 0.009 | 0.679 | 0.8998261 |
|  | Mn | 0.039 | 1.541 | -0.011 | 0.088 | 0.124 | 0.5373333 |
|  | Ni | -0.002 | -0.152 | -0.028 | 0.024 | 0.880 | 0.9533333 |
|  | Cd | 0.004 | 0.442 | -0.014 | 0.022 | 0.659 | 0.8998261 |
|  | Sn | -0.009 | -0.510 | -0.042 | 0.024 | 0.611 | 0.8998261 |
|  | Sb | -0.002 | -0.071 | -0.053 | 0.049 | 0.943 | 0.98072 |
|  | Cs | -0.047 | -2.060 | -0.092 | -0.002 | **0.040** | 0.3466667 |
|  | Ba | 0.013 | 1.437 | -0.005 | 0.031 | 0.152 | 0.5645714 |
|  | W | -0.006 | -0.431 | -0.034 | 0.022 | 0.667 | 0.8998261 |
|  | Hg | -0.007 | -0.471 | -0.036 | 0.022 | 0.638 | 0.8998261 |
|  | Tl | -0.061 | -2.812 | -0.104 | -0.019 | **0.005** | 0.13 |
|  | Pb | 0.006 | 0.387 | -0.024 | 0.035 | 0.699 | 0.8998261 |
|  | Fe | 0.006 | 0.305 | -0.034 | 0.046 | 0.760 | 0.8998261 |
|  | Co | -0.023 | -0.868 | -0.074 | 0.029 | 0.386 | 0.8998261 |
|  | Cu | -0.073 | -2.432 | -0.132 | -0.014 | **0.016** | 0.208 |
|  | Zn | -0.023 | -0.639 | -0.095 | 0.048 | 0.523 | 0.8998261 |
|  | As | 0.011 | 1.835 | -0.001 | 0.023 | 0.067 | 0.4355 |
|  | Se | -0.011 | -0.259 | -0.094 | 0.072 | 0.796 | 0.8998261 |
|  | Rb | -0.017 | -0.620 | -0.071 | 0.037 | 0.536 | 0.8998261 |
|  | Sr | -0.027 | -0.761 | -0.097 | 0.043 | 0.447 | 0.8998261 |
|  | Mo | 0.035 | 1.722 | -0.005 | 0.074 | 0.086 | 0.4472 |
|  | La | -0.004 | -0.300 | -0.030 | 0.022 | 0.764 | 0.8998261 |
|  | Ce | 0.000 | 0.012 | -0.034 | 0.035 | 0.990 | 0.99 |
|  | Pr | -0.011 | -1.010 | -0.032 | 0.010 | 0.313 | 0.8998261 |
|  | Th | -0.007 | -0.620 | -0.030 | 0.016 | 0.536 | 0.8998261 |
|  | U | -0.008 | -0.773 | -0.029 | 0.013 | 0.440 | 0.8998261 |
| Chao | Cr | -1.050 | -2.166 | -1.999 | -0.100 | **0.031** | **0.07327273** |
|  | Mn | 3.834 | 1.921 | -0.078 | 7.746 | 0.056 | 0.112 |
|  | Ni | 0.295 | 0.274 | -1.811 | 2.401 | 0.784 | 0.88616667 |
|  | Cd | 1.207 | 1.624 | -0.249 | 2.664 | 0.105 | 0.182 |
|  | Sn | 0.160 | 0.119 | -2.468 | 2.788 | 0.905 | 0.9412 |
|  | Sb | -5.348 | -2.621 | -9.347 | -1.349 | **0.009** | **0.03342857** |
|  | Cs | -1.787 | -0.979 | -5.363 | 1.790 | 0.328 | 0.47377778 |
|  | Ba | 2.221 | 3.103 | 0.818 | 3.623 | **0.002** | **0.026** |
|  | W | -2.660 | -2.393 | -4.840 | -0.481 | **0.017** | **0.0494** |
|  | Hg | -0.274 | -0.230 | -2.605 | 2.057 | 0.818 | 0.88616667 |
|  | Tl | -4.109 | -2.362 | -7.518 | -0.700 | **0.019** | **0.0494** |
|  | Pb | 2.345 | 1.971 | 0.013 | 4.676 | **0.0495** | 0.10725 |
|  | Fe | -1.156 | -0.717 | -4.320 | 2.007 | 0.474 | 0.64863158 |
|  | Co | -5.852 | -2.819 | -9.919 | -1.784 | **0.005** | **0.026** |
|  | Cu | -7.146 | -3.006 | -11.804 | -2.487 | **0.003** | **0.026** |
|  | Zn | -0.161 | -0.055 | -5.887 | 5.565 | 0.956 | 0.956 |
|  | As | 2.269 | 4.842 | 1.351 | 3.188 | **0.000002** | **0.000052** |
|  | Se | -3.708 | -1.099 | -10.322 | 2.906 | 0.273 | 0.443625 |
|  | Rb | 0.856 | 0.393 | -3.417 | 5.130 | 0.695 | 0.82136364 |
|  | Sr | -1.710 | -0.603 | -7.264 | 3.845 | 0.547 | 0.7111 |
|  | Mo | -0.730 | -0.454 | -3.880 | 2.420 | 0.650 | 0.8047619 |
|  | La | -2.980 | -2.840 | -5.036 | -0.923 | **0.005** | **0.026** |
|  | Ce | -1.469 | -1.054 | -4.198 | 1.261 | 0.292 | 0.44658824 |
|  | Pr | -2.297 | -2.670 | -3.983 | -0.611 | **0.008** | **0.03342857** |
|  | Th | -1.689 | -1.816 | -3.512 | 0.134 | 0.070 | 0.13 |
|  | U | -1.974 | -2.353 | -3.617 | -0.330 | **0.019** | **0.0494** |
| Pielou | Cr | -0.001 | -0.267 | -0.007 | 0.005 | 0.789 | 0.999 |
|  | Mn | 0.011 | 0.952 | -0.012 | 0.035 | 0.342 | 0.999 |
|  | Ni | 0.000 | -0.001 | -0.013 | 0.013 | 0.999 | 0.999 |
|  | Cd | -0.001 | -0.203 | -0.010 | 0.008 | 0.839 | 0.999 |
|  | Sn | -0.003 | -0.368 | -0.019 | 0.013 | 0.713 | 0.999 |
|  | Sb | 0.001 | 0.046 | -0.024 | 0.025 | 0.963 | 0.999 |
|  | Cs | -0.017 | -1.584 | -0.039 | 0.004 | 0.114 | 0.741 |
|  | Ba | 0.002 | 0.565 | -0.006 | 0.011 | 0.573 | 0.999 |
|  | W | 0.002 | 0.311 | -0.011 | 0.015 | 0.756 | 0.999 |
|  | Hg | -0.001 | -0.192 | -0.015 | 0.013 | 0.848 | 0.999 |
|  | Tl | -0.021 | -2.027 | -0.042 | -0.001 | **0.043** | 0.3726667 |
|  | Pb | -0.001 | -0.114 | -0.015 | 0.013 | 0.909 | 0.999 |
|  | Fe | 0.001 | 0.123 | -0.018 | 0.020 | 0.902 | 0.999 |
|  | Co | -0.004 | -0.288 | -0.028 | 0.021 | 0.774 | 0.999 |
|  | Cu | -0.032 | -2.230 | -0.060 | -0.004 | **0.026** | 0.3726667 |
|  | Zn | -0.015 | -0.867 | -0.049 | 0.019 | 0.387 | 0.999 |
|  | As | 0.003 | 1.017 | -0.003 | 0.009 | 0.310 | 0.999 |
|  | Se | 0.013 | 0.621 | -0.027 | 0.052 | 0.535 | 0.999 |
|  | Rb | -0.007 | -0.547 | -0.033 | 0.018 | 0.585 | 0.999 |
|  | Sr | -0.007 | -0.417 | -0.040 | 0.026 | 0.677 | 0.999 |
|  | Mo | 0.020 | 2.076 | 0.001 | 0.039 | **0.039** | 0.3726667 |
|  | La | 0.002 | 0.267 | -0.011 | 0.014 | 0.789 | 0.999 |
|  | Ce | 0.002 | 0.269 | -0.014 | 0.019 | 0.788 | 0.999 |
|  | Pr | -0.003 | -0.514 | -0.013 | 0.008 | 0.607 | 0.999 |
|  | Th | 0.000 | -0.051 | -0.011 | 0.011 | 0.959 | 0.999 |
|  | U | -0.001 | -0.132 | -0.011 | 0.009 | 0.895 | 0.999 |

**Table S5 Associations between ln-transformed metals and beta diversity in unadjusted and adjusted models.** *P values* (pval columns) were calculated from adonis models. False discovery rate (FDR) adjusted *q* values are shown in the FDR_qval columns. The fraction of variance explained (R2 ) is in the last column. Models were adjusted for Preterm, Sex, Antibiotic, Ever Breastfed, Delivery Mode, Birth Weight and Sample Age. q* < 0.1 was considered statistically significant.

| Metals | Unadjusted_  pval | Unadjusted_  FDR_qval | R^2^ | Adjusted  _pval | Adjusted_  FDR_qval | R^2^ |
| --- | --- | --- | --- | --- | --- | --- |
| Cr | 0.201 | 0.2678 | 0.003922 | 0.167 | 0.310142857 | 0.003916 |
| Mn | 0.056 | 0.167555556 | 0.005703 | **0.005*** | **0.043333333*** | 0.006867 |
| Ni | 0.162 | 0.252352941 | 0.004151 | 0.618 | 0.676 | 0.002339 |
| Cd | 0.067 | 0.1742 | 0.005241 | **0.044*** | 0.195 | 0.005502 |
| Sn | **0.002*** | **0.039*** | 0.011598 | 0.089 | 0.226909091 | 0.004793 |
| Sb | 0.385 | 0.455 | 0.002997 | 0.453 | 0.548363636 | 0.002681 |
| Cs | **0.041*** | 0.167555556 | 0.005815 | **0.021*** | 0.1365 | 0.006459 |
| Ba | **0.029*** | 0.1508 | 0.006118 | **0.002*** | **0.026*** | 0.009235 |
| W | 0.788 | 0.788 | 0.001897 | 0.682 | 0.705 | 0.002143 |
| Hg | **0.015*** | 0.13 | 0.006977 | 0.096 | 0.226909091 | 0.004539 |
| Tl | 0.116 | 0.232 | 0.004383 | 0.129 | 0.2795 | 0.004266 |
| Pb | 0.165 | 0.252352941 | 0.003982 | 0.06 | 0.195 | 0.005274 |
| Fe | 0.129 | 0.239571429 | 0.004242 | 0.453 | 0.548363636 | 0.002798 |
| Co | 0.466 | 0.504833333 | 0.002740 | 0.464 | 0.548363636 | 0.002597 |
| Cu | 0.058 | 0.167555556 | 0.005406 | 0.059 | 0.195 | 0.005148 |
| Zn | 0.142 | 0.246133333 | 0.004453 | 0.391 | 0.548363636 | 0.002976 |
| As | **0.003*** | **0.039*** | 0.009313 | **0.001*** | **0.026*** | 0.014100 |
| Se | **0.027*** | 0.1508 | 0.006344 | 0.705 | 0.705 | 0.002043 |
| Rb | 0.054 | 0.167555556 | 0.005181 | 0.051 | 0.195 | 0.005512 |
| Sr | 0.096 | 0.226909091 | 0.004673 | 0.278 | 0.425176471 | 0.003324 |
| Mo | 0.651 | 0.67704 | 0.002197 | 0.624 | 0.676 | 0.002211 |
| La | 0.335 | 0.414761905 | 0.003195 | 0.213 | 0.346125 | 0.003669 |
| Ce | 0.454 | 0.504833333 | 0.002850 | 0.441 | 0.548363636 | 0.002746 |
| Pr | 0.196 | 0.2678 | 0.003966 | 0.152 | 0.304 | 0.004074 |
| Th | 0.113 | 0.232 | 0.004442 | 0.068 | 0.196444444 | 0.004927 |
| U | 0.206 | 0.2678 | 0.003774 | 0.183 | 0.3172 | 0.003829 |

**Table S6 Unadjusted taxa associations from 16S rRNA gene amplicon sequencing.** The effect size estimate expressed in the coef column represents the changes in species relative abundance (ln transformed) per unit increase in blood metals. Associations were analyzed using MaAsLin2. False discovery rate (FDR) adjusted *q* values are in the last column (qval).

| Metals | Phylum | genus | Coef | SE | p-Value | FDR_q-Value |
| --- | --- | --- | --- | --- | --- | --- |
| Cr | *Bacteroidota* |  | -0.935228774 | 0.226073895 | 4.44E-05 | 0.000488713 |
| Cr | *Actinobacteriota* |  | -0.502450938 | 0.180831015 | 0.005763229 | 0.031697758 |
| Mn | *Bacteroidota* |  | 0.648550297 | 0.229008306 | 0.004901477 | 0.053916248 |
| Cd | *Bacteroidota* |  | 0.664041845 | 0.228877692 | 0.003958372 | 0.043542097 |
| Cs | *Gemmatimonadota* |  | 0.178038998 | 0.055617199 | 0.00149792 | 0.01647712 |
| Ba | *Bacteroidota* |  | 0.719795605 | 0.228381422 | 0.00176739 | 0.009720645 |
| Ba | *Deinococcota* |  | 0.148978737 | 0.053552838 | 0.005705654 | 0.02092073 |
| W | *Actinobacteriota* |  | -0.584186597 | 0.180107272 | 0.001297605 | 0.013958902 |
| W | *Deinococcota* |  | -0.1625217 | 0.053436866 | 0.002537982 | 0.013958902 |
| Tl | *Bacteroidota* |  | -0.580818554 | 0.229542388 | 0.011846237 | 0.050322146 |
| Tl | *Deinococcota* |  | -0.132972156 | 0.053676626 | 0.013724222 | 0.050322146 |
| Tl | *Gemmatimonadota* |  | 0.140287272 | 0.05593405 | 0.012604045 | 0.050322146 |
| Co | *Actinobacteriota* |  | -0.540788749 | 0.180505463 | 0.002936668 | 0.032303343 |
| As | *Bacteroidota* |  | 1.098853527 | 0.223898426 | 1.43E-06 | 1.57E-05 |
| As | *Actinobacteriota* |  | 0.567506712 | 0.180264056 | 0.001788248 | 0.00655691 |
| As | *Deinococcota* |  | 0.15999012 | 0.053459331 | 0.002967053 | 0.008159395 |
| As | *Proteobacteria* |  | 0.510665793 | 0.189391435 | 0.00735928 | 0.016190416 |
| As | *Firmicutes* |  | -0.216904633 | 0.086499908 | 0.012622011 | 0.023140353 |
| As | *Planctomycetota* |  | 0.093401448 | 0.043044116 | 0.030706443 | 0.048252982 |
| La | *Bacteroidota* |  | -0.742587267 | 0.228166703 | 0.001249709 | 0.006873398 |
| Pr | *Bacteroidota* |  | -0.565606227 | 0.229654091 | 0.01427839 | 0.098394864 |
| Cr | *Proteobacteria* | *Burkholderia.Caballeronia.Paraburkholderia* | 0.427710379 | 0.095401179 | 1.01E-05 | 0.000332055 |
| Cr | *Firmicutes* | *Lactobacillus* | -0.53479737 | 0.136843364 | 0.000112271 | 0.001852478 |
| Cr | *Proteobacteria* | *Ralstonia* | 0.260499195 | 0.071175923 | 0.000292357 | 0.003215923 |
| Cr | *Firmicutes* | *Lactococcus* | -0.382655114 | 0.107431472 | 0.000420781 | 0.003471442 |
| Cr | *Bacteroidota* | *Bacteroides* | -0.657036233 | 0.21786618 | 0.00275616 | 0.018190654 |
| Cr | *Actinobacteriota* | *Rothia* | -0.513984391 | 0.193874106 | 0.008397891 | 0.046188399 |
| Cr | *Firmicutes* | *Streptococcus* | -0.360913293 | 0.144112108 | 0.012733847 | 0.060030992 |
| Mn | *Firmicutes* | *Enterococcus* | -0.683288394 | 0.168061469 | 5.95E-05 | 0.000982487 |
| Mn | *Proteobacteria* | *Burkholderia.Caballeronia.Paraburkholderia* | -0.402426738 | 0.095724171 | 3.36E-05 | 0.000982487 |
| Mn | *Proteobacteria* | *Ralstonia* | -0.232569801 | 0.071459886 | 0.001249844 | 0.013748282 |
| Sn | *Actinobacteriota* | *Bifidobacterium* | -1.026357641 | 0.227206634 | 8.65E-06 | 0.00028553 |
| Sn | *Proteobacteria* | *Acinetobacter* | 0.603707041 | 0.163258649 | 0.000253416 | 0.004181369 |
| Sn | *Proteobacteria* | *Pseudomonas* | 0.421325512 | 0.143694056 | 0.003594927 | 0.029658144 |
| Sn | *Proteobacteria* | *Methylobacterium.Methylorubrum* | 0.177657273 | 0.05955015 | 0.003057506 | 0.029658144 |
| Sn | *Firmicutes* | *Veillonella* | -0.659080392 | 0.24035262 | 0.006427103 | 0.035349064 |
| Sn | *Proteobacteria* | *Stenotrophomonas* | 0.261344613 | 0.094211296 | 0.005842434 | 0.035349064 |
| Sn | *Actinobacteriota* | *Corynebacterium* | 0.459588047 | 0.173128074 | 0.008313324 | 0.039191383 |
| Sn | *Bacteroidota* | *Spirosoma* | 0.174543587 | 0.074602277 | 0.019879812 | 0.079873864 |
| Sn | *Proteobacteria* | *Brevundimonas* | 0.144082781 | 0.062516163 | 0.021783781 | 0.079873864 |
| Ba | *Proteobacteria* | *Acinetobacter* | 0.767912455 | 0.1612171 | 2.83E-06 | 6.63E-05 |
| Ba | *Proteobacteria* | *Burkholderia.Caballeronia.Paraburkholderia* | -0.446001746 | 0.095154511 | 4.02E-06 | 6.63E-05 |
| Ba | *Proteobacteria* | *Ralstonia* | -0.27198782 | 0.071049414 | 0.000153671 | 0.001690377 |
| Ba | *Firmicutes* | *Enterococcus* | -0.544514583 | 0.16954585 | 0.001446017 | 0.011929644 |
| Ba | *Proteobacteria* | *Pseudomonas* | 0.383381071 | 0.144006209 | 0.008130771 | 0.053663091 |
| Ba | *Actinobacteriota* | *Bifidobacterium* | -0.589623203 | 0.23172959 | 0.011386852 | 0.053680876 |
| Ba | *Deinococcota* | *Thermus* | 0.11382444 | 0.044488505 | 0.01094478 | 0.053680876 |
| Ba | *Firmicutes* | *Lactobacillus* | 0.319836356 | 0.138803597 | 0.021811685 | 0.079976178 |
| W | *Proteobacteria* | *Burkholderia.Caballeronia.Paraburkholderia* | 0.38108658 | 0.095980698 | 8.76E-05 | 0.002889873 |
| W | *Actinobacteriota* | *Rothia* | -0.622970569 | 0.192931907 | 0.001363515 | 0.022497997 |
| W | *Firmicutes* | *Lacticaseibacillus* | -0.359564094 | 0.134751122 | 0.007987552 | 0.03294865 |
| W | *Proteobacteria* | *Ralstonia* | 0.199600083 | 0.07175251 | 0.005707654 | 0.03294865 |
| W | *Firmicutes* | *Finegoldia* | -0.237575243 | 0.086364742 | 0.006262614 | 0.03294865 |
| W | *Bacteroidota* | *Spirosoma* | -0.201875132 | 0.074399197 | 0.006998725 | 0.03294865 |
| W | *Deinococcota* | *Thermus* | -0.131885887 | 0.044341566 | 0.003146302 | 0.03294865 |
| W | *Proteobacteria* | *Pseudomonas* | -0.35507063 | 0.144219541 | 0.014311541 | 0.05247565 |
| W | *Proteobacteria* | *Acinetobacter* | -0.383316811 | 0.165206483 | 0.020919975 | 0.069035917 |
| W | *Firmicutes* | *Veillonella* | -0.529380106 | 0.241293899 | 0.028917329 | 0.086751986 |
| Tl | *Proteobacteria* | *Burkholderia.Caballeronia.Paraburkholderia* | 0.368590313 | 0.096124127 | 0.000149902 | 0.00494675 |
| Tl | *Bacteroidota* | *Spirosoma* | -0.237121752 | 0.074092721 | 0.001502022 | 0.024783359 |
| Pb | *Proteobacteria* | *Ralstonia* | -0.382890661 | 0.069529906 | 7.22E-08 | 2.38E-06 |
| Pb | *Proteobacteria* | *Burkholderia.Caballeronia.Paraburkholderia* | -0.453140923 | 0.095055253 | 2.78E-06 | 4.58E-05 |
| Pb | *Proteobacteria* | *Pseudomonas* | 0.593675107 | 0.14189245 | 3.65E-05 | 0.000401561 |
| Pb | *Proteobacteria* | *Acinetobacter* | 0.654779454 | 0.162678657 | 7.03E-05 | 0.000579832 |
| Pb | *Actinobacteriota* | *Rothia* | 0.492162993 | 0.194040573 | 0.011646999 | 0.076870197 |
| Co | *Firmicutes* | *Lactobacillus* | -0.379032824 | 0.138364592 | 0.006480156 | 0.071281721 |
| Co | *Firmicutes* | *Finegoldia* | -0.247192044 | 0.086285324 | 0.004431858 | 0.071281721 |
| Cu | *Proteobacteria* | *Acinetobacter* | -0.478712868 | 0.164472844 | 0.003845345 | 0.062989747 |
| Cu | *Actinobacteriota* | *Corynebacterium* | -0.501641576 | 0.172784371 | 0.003933975 | 0.062989747 |
| Cu | *Proteobacteria* | *Methylobacterium.Methylorubrum* | -0.165868941 | 0.059650071 | 0.005726341 | 0.062989747 |
| Cu | *Firmicutes* | *Staphylococcus* | -0.534841185 | 0.228056535 | 0.019590673 | 0.098290025 |
| Cu | *Firmicutes* | *Clostridium_sensu_stricto_1* | -0.559014758 | 0.256179799 | 0.029784856 | 0.098290025 |
| Cu | *Proteobacteria* | *Pseudomonas* | -0.350273483 | 0.14425404 | 0.015693467 | 0.098290025 |
| Cu | *Proteobacteria* | *Stenotrophomonas* | -0.206721448 | 0.094609545 | 0.02957137 | 0.098290025 |
| Cu | *Actinobacteriota* | *Cutibacterium* | -0.292687056 | 0.133163416 | 0.028626077 | 0.098290025 |
| Cu | *Firmicutes* | *Finegoldia* | -0.204107744 | 0.086616079 | 0.019016632 | 0.098290025 |
| Cu | *Proteobacteria* | *Brevundimonas* | -0.138161165 | 0.062555466 | 0.027867821 | 0.098290025 |
| As | *Proteobacteria* | *Burkholderia.Caballeronia.Paraburkholderia* | -0.65730679 | 0.091480548 | 4.27E-12 | 1.41E-10 |
| As | *Proteobacteria* | *Acinetobacter* | 0.839467063 | 0.160164517 | 2.81E-07 | 3.09E-06 |
| As | *Proteobacteria* | *Ralstonia* | -0.367849364 | 0.06976833 | 2.40E-07 | 3.09E-06 |
| As | *Firmicutes* | *Enterococcus* | -0.668847202 | 0.168232244 | 8.57E-05 | 0.000565842 |
| As | *Actinobacteriota* | *Rothia* | 0.706916834 | 0.192079074 | 0.00027075 | 0.001489126 |
| As | *Firmicutes* | *Veillonella* | 0.723813304 | 0.239804278 | 0.002733491 | 0.009436354 |
| As | *Bacteroidota* | *Bacteroides* | 0.655779613 | 0.217877315 | 0.002809021 | 0.009436354 |
| As | *Bacteroidota* | *Parabacteroides* | 0.499226394 | 0.169407292 | 0.003431402 | 0.009436354 |
| As | *Proteobacteria* | *Pseudomonas* | 0.42437331 | 0.143667675 | 0.003356941 | 0.009436354 |
| As | *Bacteroidota* | *Spirosoma* | 0.221047075 | 0.074238755 | 0.003114494 | 0.009436354 |
| As | *Proteobacteria* | *Brevundimonas* | 0.185143082 | 0.062197359 | 0.003122547 | 0.009436354 |
| As | *Deinococcota* | *Thermus* | 0.111088179 | 0.044508843 | 0.013038201 | 0.033096972 |
| As | *Firmicutes* | *Lactococcus* | 0.255454465 | 0.108536862 | 0.019160387 | 0.045163769 |
| As | *Firmicutes* | *Streptococcus* | 0.327172362 | 0.144348827 | 0.024046217 | 0.049595323 |
| As | *Firmicutes* | *Bacillus* | 0.217737856 | 0.097669812 | 0.026445081 | 0.051334568 |
| As | *Firmicutes* | *Staphylococcus* | -0.492382119 | 0.228337606 | 0.031754997 | 0.058217495 |
| As | *Proteobacteria* | *Sphingomonas* | 0.114904105 | 0.056123856 | 0.041393764 | 0.071894433 |
| As | *Firmicutes* | *Lactobacillus* | 0.277949653 | 0.139068629 | 0.046441954 | 0.076629223 |
| Se | *Firmicutes* | *Staphylococcus* | -0.950812386 | 0.224036087 | 2.84E-05 | 0.000545714 |
| Se | *Proteobacteria* | *Acinetobacter* | -0.683034186 | 0.162336596 | 3.31E-05 | 0.000545714 |
| Se | *Actinobacteriota* | *Bifidobacterium* | 0.840917376 | 0.229436897 | 0.000286701 | 0.003153708 |
| Se | *Proteobacteria* | *Methylobacterium.Methylorubrum* | -0.203947832 | 0.059301878 | 0.000656118 | 0.005412976 |
| Se | *Deinococcota* | *Thermus* | -0.122096999 | 0.044423944 | 0.006307356 | 0.04162855 |
| Se | *Proteobacteria* | *Pseudomonas* | -0.367528236 | 0.144127721 | 0.011209874 | 0.061654308 |
| Sr | *Firmicutes* | *Staphylococcus* | -0.716600577 | 0.226585038 | 0.001704453 | 0.030027314 |
| Sr | *Proteobacteria* | *Stenotrophomonas* | -0.295173867 | 0.093916963 | 0.001819837 | 0.030027314 |
| Mo | *Proteobacteria* | *Burkholderia.Caballeronia.Paraburkholderia* | -0.329375371 | 0.096541959 | 0.000723324 | 0.023869695 |
| La | *Proteobacteria* | *Burkholderia.Caballeronia.Paraburkholderia* | 0.440891151 | 0.095224534 | 5.21E-06 | 0.000171976 |
| La | *Proteobacteria* | *Acinetobacter* | -0.590092051 | 0.163404991 | 0.000350661 | 0.005785907 |
| La | *Bacteroidota* | *Spirosoma* | -0.239521236 | 0.074070017 | 0.001341722 | 0.014758945 |
| La | *Actinobacteriota* | *Rothia* | -0.601493452 | 0.193132254 | 0.001999752 | 0.016497953 |
| La | *Proteobacteria* | *Methylobacterium.Methylorubrum* | -0.162538609 | 0.059677029 | 0.006790101 | 0.037345555 |
| La | *Proteobacteria* | *Ralstonia* | 0.165734132 | 0.072005639 | 0.021957139 | 0.090573197 |
| Pr | *Proteobacteria* | *Burkholderia.Caballeronia.Paraburkholderia* | 0.41454373 | 0.095571971 | 1.90E-05 | 0.00062764 |
| Pr | *Proteobacteria* | *Acinetobacter* | -0.613514919 | 0.163151076 | 0.000199664 | 0.003294457 |
| Pr | *Actinobacteriota* | *Rothia* | -0.569270031 | 0.193419302 | 0.003471732 | 0.028641787 |
| Pr | *Firmicutes* | *Veillonella* | -0.662779984 | 0.240322697 | 0.006132273 | 0.040473005 |
| Pr | *Bacteroidota* | *Spirosoma* | -0.190592811 | 0.07448667 | 0.010937463 | 0.051562325 |
| Pr | *Proteobacteria* | *Methylobacterium.Methylorubrum* | -0.155216383 | 0.059734336 | 0.009772295 | 0.051562325 |
| Pr | *Proteobacteria* | *Sphingomonas* | -0.139285245 | 0.055961232 | 0.013289549 | 0.05481939 |
| Pr | *Proteobacteria* | *Ralstonia* | 0.165456144 | 0.072007519 | 0.022182063 | 0.073200808 |
| Th | *Proteobacteria* | *Acinetobacter* | -0.687842819 | 0.162276868 | 2.90E-05 | 0.000956965 |
| Th | *Actinobacteriota* | *Rothia* | -0.682737701 | 0.192336154 | 0.000439938 | 0.004515837 |
| Th | *Proteobacteria* | *Burkholderia.Caballeronia.Paraburkholderia* | 0.3366378 | 0.096468252 | 0.000547374 | 0.004515837 |
| Th | *Proteobacteria* | *Sphingomonas* | -0.197900372 | 0.055439422 | 0.000408867 | 0.004515837 |
| Th | *Proteobacteria* | *Methylobacterium.Methylorubrum* | -0.153411206 | 0.059748051 | 0.010666395 | 0.058665175 |

**Table S7 Adjusted taxa associations from 16S rRNA gene amplicon sequencing.** The effect size estimate expressed in the coef column represents the changes in species relative abundance (ln transformed) per unit increase in blood metals. Associations were analyzed using MaAsLin2. False discovery rate (FDR) adjusted *q* values are in the last column (qval). Models were adjusted for Preterm, Sex, Antibiotic, Ever Breastfed, Delivery Mode, Birth Weight and Sample Age

| Metals | Phylum | genus | Coef | SE | p-Value | FDR_q-Value |
| --- | --- | --- | --- | --- | --- | --- |
| Cr | *Firmicutes* | *Lactobacillus* | -0.513599963 | 0.14136094 | 0.000324009 | 0.014256388 |
| Cr | *Proteobacteria* | *Burkholderia.Caballeronia.Paraburkholderia* | 0.322498197 | 0.096423818 | 0.000918007 | 0.030294228 |
| Cr | *Proteobacteria* | *Ralstonia* | 0.217112166 | 0.07514915 | 0.004116796 | 0.077631011 |
| Mn | *Firmicutes* | *Enterococcus* | -0.695522154 | 0.176244559 | 9.68E-05 | 0.005112158 |
| Mn | *Proteobacteria* | *Ralstonia* | -0.242927743 | 0.074977245 | 0.001315829 | 0.041514038 |
| Mn | *Proteobacteria* | *Burkholderia.Caballeronia.Paraburkholderia* | -0.310981087 | 0.096622071 | 0.001415251 | 0.041514038 |
| Mn | *Firmicutes* | *Clostridium_sensu_stricto_1* | 0.769444877 | 0.258495408 | 0.003127759 | 0.058980596 |
| Cd | *Proteobacteria* | *Acinetobacter* | 0.47544061 | 0.16165573 | 0.003499803 | 0.083995282 |
| Ba | *Firmicutes* | *Enterococcus* | -0.683243138 | 0.182201031 | 0.000208526 | 0.009175124 |
| Ba | *Proteobacteria* | *Acinetobacter* | 0.601358433 | 0.171784975 | 0.000527339 | 0.015808763 |
| Ba | *Proteobacteria* | *Ralstonia* | -0.267418152 | 0.077173587 | 0.000599279 | 0.015820963 |
| Ba | *Firmicutes* | *Clostridium_sensu_stricto_1* | 0.773265728 | 0.26683207 | 0.004005016 | 0.081332634 |
| W | *Firmicutes* | *Finegoldia* | -0.250439956 | 0.086838563 | 0.004182533 | 0.096678631 |
| W | *Proteobacteria* | *Burkholderia.Caballeronia.Paraburkholderia* | 0.259918757 | 0.095069357 | 0.006591725 | 0.096678631 |
| Tl | *Proteobacteria* | *Burkholderia.Caballeronia.Paraburkholderia* | 0.280017981 | 0.096497261 | 0.003957045 | 0.094969072 |
| Pb | *Proteobacteria* | *Ralstonia* | -0.387634731 | 0.072880343 | 1.92E-07 | 5.07E-05 |
| Pb | *Proteobacteria* | *Pseudomonas* | 0.551007826 | 0.149056829 | 0.000255395 | 0.009632038 |
| Pb | *Proteobacteria* | *Burkholderia.Caballeronia.Paraburkholderia* | -0.305497092 | 0.096364767 | 0.001664715 | 0.048831627 |
| Pb | *Proteobacteria* | *Acinetobacter* | 0.497665604 | 0.166824959 | 0.003063495 | 0.062212522 |
| Cu | *Firmicutes* | *Finegoldia* | -0.339650303 | 0.09704783 | 0.00052891 | 0.017454031 |
| Cu | *Actinobacteriota* | *Cutibacterium* | -0.536039191 | 0.14930603 | 0.000380123 | 0.014336083 |
| Cu | *Firmicutes* | *Lactobacillus* | -0.430230555 | 0.157107644 | 0.00650508 | 0.095407837 |
| Cu | *Firmicutes* | *Clostridium_sensu_stricto_1* | -0.767086965 | 0.285355208 | 0.007545348 | 0.098573557 |
| As | *Proteobacteria* | *Ralstonia* | -0.393329968 | 0.078649454 | 9.24E-07 | 8.13E-05 |
| As | *Proteobacteria* | *Burkholderia.Caballeronia.Paraburkholderia* | -0.498857654 | 0.101451839 | 1.38E-06 | 9.12E-05 |
| As | *Proteobacteria* | *Acinetobacter* | 0.817720583 | 0.175974896 | 4.86E-06 | 0.000256788 |
| As | *Firmicutes* | *Veillonella* | 1.138172248 | 0.253424449 | 9.78E-06 | 0.000368815 |
| As | *Firmicutes* | *Enterococcus* | -0.83455146 | 0.187611154 | 1.18E-05 | 0.00038986 |
| As | *Firmicutes* | *Clostridium_sensu_stricto_1* | 0.880111251 | 0.276305733 | 0.001582878 | 0.026117491 |
| As | *Proteobacteria* | *Pseudomonas* | 0.437295876 | 0.161609772 | 0.007163205 | 0.090051726 |
| Se | *Firmicutes* | *Staphylococcus* | -0.913784242 | 0.267174575 | 0.000703628 | 0.026414159 |
| Se | *Deinococcota* | *Thermus* | -0.16027991 | 0.052725238 | 0.002553999 | 0.074917307 |
| La | *Proteobacteria* | *Acinetobacter* | -0.499559635 | 0.165926646 | 0.002805389 | 0.079894618 |
| La | *Proteobacteria* | *Burkholderia.Caballeronia.Paraburkholderia* | 0.28752354 | 0.096020838 | 0.002955858 | 0.079894618 |
| La | *Proteobacteria* | *Methylobacterium.Methylorubrum* | -0.166616815 | 0.061700098 | 0.007278909 | 0.096081605 |
| Pr | *Proteobacteria* | *Burkholderia.Caballeronia.Paraburkholderia* | 0.311497942 | 0.093692195 | 0.00098379 | 0.037102924 |
| Pr | *Proteobacteria* | *Acinetobacter* | -0.495292422 | 0.162338126 | 0.002464069 | 0.064627127 |
| Pr | *Firmicutes* | *Veillonella* | -0.64808682 | 0.233873962 | 0.005900651 | 0.091633641 |
| Th | *Proteobacteria* | *Acinetobacter* | -0.56239165 | 0.160603944 | 0.000525275 | 0.019810372 |
| Th | *Proteobacteria* | *Sphingomonas* | -0.185509238 | 0.056772902 | 0.001197842 | 0.039528797 |
| Th | *Actinobacteriota* | *Rothia* | -0.594101861 | 0.188113676 | 0.001732733 | 0.045744145 |
| Th | *Proteobacteria* | *Burkholderia.Caballeronia.Paraburkholderia* | 0.289141 | 0.093286698 | 0.002103811 | 0.050491473 |

**Table S8** **Posterior Inclusion Probabilities (PIPs) for ln-transformed metals selected by single-metal generalized linear models (GLMs) or Elastic Net (EN) regression, associated with indices of alpha diversity.** Models were adjusted for Preterm, Sex, Antibiotic, Ever Breastfed, Delivery Mode, Birth Weight and Sample Age. Variables in bold represent those with PIP > 0.7

| Alpha Diversity | **GLMs** | |  | **EN** | |
| --- | --- | --- | --- | --- | --- |
|  | Metals | PIP |  | Metals | PIP |
| Chao | Cr | 0.5634 |  | Cd | 0.5656 |
| Chao | **Sb** | **0.8764** |  | **Sb** | **0.9506** |
| Chao | Ba | 0.4594 |  | Ba | 0.4026 |
| Chao | W | 0.4932 |  | W | 0.4542 |
| Chao | Tl | 0.2938 |  | Co | 0.6262 |
| Chao | Co | 0.6396 |  | **Cu** | **0.7236** |
| Chao | Cu | 0.6678 |  | **As** | **0.9982** |
| Chao | **As** | **0.9954** |  | **U** | **0.9732** |
| Chao | La | 0.436 |  |  |  |
| Chao | Pr | 0.5124 |  |  |  |
| Chao | **U** | **0.8892** |  |  |  |

**Table S9 Taxa associated with multiple metals and Posterior Inclusion Probabilities values (PIPs) of ln-transformed metals in the BKMR model.** Models were adjusted for Preterm, Sex, Antibiotic, Ever Breastfed, Delivery Mode, Birth Weight and Sample Age. Variables in bold represent those with PIP > 0.7

| genus | Metals | PIP |
| --- | --- | --- |
| *Burkholderia.Caballeronia.Paraburkholderia* | Cr | 0 |
|  | Mn | 0.5348 |
|  | W | 0.0142 |
|  | Tl | 0 |
|  | Pb | 0 |
|  | As | 0.034 |
|  | La | 0.004 |
|  | Pr | 0 |
|  | Th | 0.005 |
| *Acinetobacter* | Cr | 0.0338 |
|  | Ba | 0.002 |
|  | Pb | 0.0504 |
|  | As | 0.0226 |
|  | La | 0.0362 |
|  | Pr | 0.066 |
|  | Th | 0.0782 |
| *Ralstonia* | Cr | 0.006 |
|  | Mn | 0 |
|  | Ba | 0.0046 |
|  | Pb | 0.0054 |
|  | **As** | **0.8858** |
| *Clostridium_sensu_stricto_1* | **Mn** | **0.7656** |
|  | Ba | 0.6704 |
|  | **Cu** | **0.867** |
|  | As | 0.6386 |
| *Enterococcus* | Mn | 0.3892 |
|  | Ba | 0.093 |
|  | As | 0.3166 |

**Table S10. Associations between WQS index and Chao or genus by WQS regression model in the positive and negative directions.** Models were adjusted for Gestational Age, Sex, Antibiotic, Ever Breastfed, Delivery Mode, Birth Weight and Sample Age. Boldface and asterisks (*) denote metals with p-values < 0.05; the metal column presents the most heavily weighted metals (with weights) in positive/negative associations.

| Outcome | Direction | Adjusted model | | Metal（weight） |
| --- | --- | --- | --- | --- |
|  |  | β- value | *p-*value |  |
| Chao_ GLMs | Positive | 5.79 | **0.002*** | **As (0.589)** |
|  | Negative | -8.20107 | **0.000055*** | **Cu (0.27)** |
| Chao_ EN | Positive | 6.104574 | **0.0000833*** | **As (0.653)** |
|  | Negative | -5.099 | **0.00509*** | **Cu (0.361)** |
| *Burkholderia.Caballeronia.*  *Paraburkholderia* | Positive | -0.294 | 0.158 | As (0.368) |
|  | Negative | 0.077 | 0.6487 | Cr (0.552) |
| *Acinetobacter* | Positive | 0.239 | 0.372 | Cd (0.617) |
|  | Negative | -0.434 | 0.187 | Pr (0.372) |
| *Ralstonia* | Positive | 0.103 | 0.325 | As (0.594) |
|  | Negative | -0.0806 | 0.552 | Cr (0.59) |
| *Clostridium_sensu_stricto_1* | Positive | -0.219811 | 0.643 | As (0.654) |
|  | Negative | 0.654 | 0.167 | Ba (0.485) |
| *Ralstonia* | Positive | -0.03996 | 0.85469 | As (0.853) |
|  | Negative | -0.109292 | 0.61819 | Mn (0.722) |

Sensitivity analysis—3groups

**Table S11 Undjusted associations of ln-transformed metals with alpha diversity, stratified by preterm(≥37 is considered non-preterm, < 37 and≥32 is preterm，<32 is very preterm).** The effect size estimate expressed in the coef column represents the changes in species relative abundance per unit increase in blood metals. *P* value (pval column) calculated from Generalized linear regression. Bold*: q < 0.01.

| Method | Metals | Non-preterm=95 | | Preterm =191 | | Very preterm=56 | |
| --- | --- | --- | --- | --- | --- | --- | --- |
|  |  | coef | pval | coef | pval | coef | pval |
| Shannon | Cr | -0.013 | 0.597 | 0.017 | 0.490 | -0.081 | 0.054 |
|  | Mn | 0.221 | 0.075 | 0.028 | 0.736 | -0.230 | 0.249 |
|  | Ni | 0.026 | 0.594 | -0.094 | 0.113 | 0.039 | 0.696 |
|  | Cd | 0.037 | 0.590 | 0.003 | 0.933 | -0.022 | 0.723 |
|  | Sn | -0.002 | 0.980 | -0.109 | 0.022 | 0.016 | 0.862 |
|  | Sb | 0.247 | 0.110 | -0.074 | 0.394 | -0.380 | 0.015 |
|  | Cs | -0.034 | 0.805 | -0.072 | 0.352 | -0.302 | 0.043 |
|  | Ba | 0.066 | 0.039 | -0.028 | 0.399 | -0.006 | 0.948 |
|  | W | 0.031 | 0.638 | -0.040 | 0.390 | -0.008 | 0.955 |
|  | Hg | -0.073 | 0.419 | -0.060 | 0.207 | -0.023 | 0.811 |
|  | Tl | -0.030 | 0.795 | -0.163 | 0.033 | -0.140 | 0.386 |
|  | Pb | 0.155 | 0.030 | -0.055 | 0.294 | -0.054 | 0.587 |
|  | Fe | -0.012 | 0.897 | 0.000 | 0.997 | -0.346 | 0.014 |
|  | Co | -0.059 | 0.588 | -0.137 | 0.163 | -0.221 | 0.291 |
|  | Cu | -0.085 | 0.538 | -0.104 | 0.252 | -0.365 | 0.086 |
|  | Zn | 0.337 | 0.116 | -0.106 | 0.426 | -0.438 | 0.046 |
|  | As | 0.040 | 0.078 | 0.001 | 0.977 | -0.002 | 0.962 |
|  | Se | 0.150 | 0.502 | 0.164 | 0.186 | -0.246 | 0.297 |
|  | Rb | 0.003 | 0.988 | -0.044 | 0.637 | -0.167 | 0.353 |
|  | Sr | 0.314 | 0.093 | -0.105 | 0.354 | 0.000 | 1.000 |
|  | Mo | 0.058 | 0.508 | 0.032 | 0.666 | 0.197 | 0.200 |
|  | La | -0.069 | 0.261 | 0.008 | 0.875 | 0.013 | 0.879 |
|  | Ce | 0.046 | 0.632 | -0.022 | 0.717 | -0.096 | 0.425 |
|  | Pr | -0.074 | 0.217 | -0.017 | 0.665 | -0.039 | 0.597 |
|  | Th | -0.035 | 0.616 | 0.002 | 0.961 | -0.030 | 0.704 |
|  | U | -0.015 | 0.821 | -0.021 | 0.549 | -0.040 | 0.572 |
| Simpson | Cr | -0.001 | 0.958 | 0.006 | 0.490 | -0.018 | 0.194 |
|  | Mn | 0.079 | 0.113 | 0.027 | 0.387 | -0.129 | 0.043 |
|  | Ni | 0.006 | 0.736 | -0.031 | 0.168 | -0.010 | 0.756 |
|  | Cd | 0.001 | 0.980 | 0.004 | 0.746 | -0.010 | 0.620 |
|  | Sn | -0.008 | 0.790 | -0.033 | 0.065 | -0.013 | 0.675 |
|  | Sb | 0.092 | 0.137 | -0.021 | 0.512 | -0.102 | 0.044 |
|  | Cs | 0.000 | 0.998 | -0.037 | 0.197 | -0.119 | 0.012 |
|  | Ba | 0.025 | 0.057 | -0.009 | 0.453 | -0.001 | 0.969 |
|  | W | 0.011 | 0.682 | -0.015 | 0.404 | 0.005 | 0.920 |
|  | Hg | -0.030 | 0.400 | -0.029 | 0.106 | 0.001 | 0.970 |
|  | Tl | -0.014 | 0.754 | -0.075 | 0.010 | -0.041 | 0.428 |
|  | Pb | 0.048 | 0.091 | -0.014 | 0.471 | -0.034 | 0.281 |
|  | Fe | 0.005 | 0.879 | 0.009 | 0.753 | -0.100 | 0.028 |
|  | Co | -0.006 | 0.883 | -0.054 | 0.147 | -0.047 | 0.492 |
|  | Cu | -0.024 | 0.669 | -0.044 | 0.205 | -0.008 | 0.908 |
|  | Zn | 0.106 | 0.216 | -0.015 | 0.773 | -0.185 | 0.008 |
|  | As | 0.015 | 0.099 | -0.003 | 0.684 | -0.009 | 0.577 |
|  | Se | 0.028 | 0.754 | 0.029 | 0.541 | -0.047 | 0.535 |
|  | Rb | 0.001 | 0.989 | -0.030 | 0.392 | -0.070 | 0.223 |
|  | Sr | 0.125 | 0.094 | -0.066 | 0.123 | 0.083 | 0.346 |
|  | Mo | 0.030 | 0.399 | 0.009 | 0.743 | 0.068 | 0.168 |
|  | La | -0.021 | 0.406 | 0.009 | 0.627 | 0.008 | 0.778 |
|  | Ce | 0.022 | 0.568 | -0.007 | 0.771 | -0.020 | 0.613 |
|  | Pr | -0.022 | 0.361 | -0.004 | 0.777 | -0.010 | 0.667 |
|  | Th | -0.004 | 0.879 | -0.003 | 0.853 | -0.003 | 0.914 |
|  | U | -0.012 | 0.629 | -0.008 | 0.543 | -0.011 | 0.621 |
| Chao | Cr | -0.666 | 0.427 | -1.029 | 0.118 | -2.958 | 0.011 |
|  | Mn | 7.733 | 0.072 | 3.475 | 0.126 | 3.714 | 0.508 |
|  | Ni | 2.405 | 0.146 | -2.750 | 0.093 | 1.529 | 0.584 |
|  | Cd | 0.544 | 0.820 | 1.298 | 0.127 | 2.165 | 0.207 |
|  | Sn | 2.277 | 0.392 | -1.261 | 0.340 | 2.858 | 0.270 |
|  | Sb | -1.495 | 0.781 | -1.989 | 0.404 | -14.461 | **0.0006*** |
|  | Cs | -2.378 | 0.620 | -1.246 | 0.557 | -4.627 | 0.272 |
|  | Ba | 2.457 | 0.027 | 2.529 | **0.005*** | 2.276 | 0.367 |
|  | W | -1.495 | 0.505 | -3.413 | **0.008*** | -6.215 | 0.107 |
|  | Hg | -1.814 | 0.561 | 0.579 | 0.660 | -2.081 | 0.441 |
|  | Tl | -10.511 | **0.007*** | -2.470 | 0.243 | -4.224 | 0.349 |
|  | Pb | 5.487 | 0.026 | 2.808 | 0.052 | 0.259 | 0.926 |
|  | Fe | -0.728 | 0.814 | 0.190 | 0.927 | -6.666 | 0.093 |
|  | Co | -7.254 | 0.052 | -4.420 | 0.103 | -9.939 | 0.087 |
|  | Cu | -3.855 | 0.418 | -4.600 | 0.066 | -13.124 | 0.026 |
|  | Zn | 5.845 | 0.432 | 0.894 | 0.807 | -6.785 | 0.275 |
|  | As | 2.089 | **0.008*** | 2.222 | **0.00003*** | 2.327 | 0.110 |
|  | Se | -10.484 | 0.174 | 1.806 | 0.598 | -9.391 | 0.152 |
|  | Rb | 2.840 | 0.631 | 1.963 | 0.442 | -4.838 | 0.336 |
|  | Sr | 7.147 | 0.270 | -1.730 | 0.580 | -3.777 | 0.621 |
|  | Mo | -2.016 | 0.507 | 0.603 | 0.765 | 1.560 | 0.718 |
|  | La | -5.429 | **0.010*** | -2.917 | 0.027 | -2.150 | 0.372 |
|  | Ce | -3.938 | 0.238 | -0.792 | 0.643 | -1.857 | 0.583 |
|  | Pr | -6.457 | **0.001*** | -1.842 | 0.079 | -1.207 | 0.555 |
|  | Th | -3.938 | 0.102 | -0.950 | 0.393 | -2.693 | 0.218 |
|  | U | -6.764 | **0.002*** | -0.297 | 0.755 | -2.906 | 0.141 |
| Pielou | Cr | -0.001 | 0.898 | 0.005 | 0.222 | -0.009 | 0.222 |
|  | Mn | 0.033 | 0.164 | 0.002 | 0.893 | -0.060 | 0.068 |
|  | Ni | 0.001 | 0.879 | -0.014 | 0.193 | 0.002 | 0.891 |
|  | Cd | 0.006 | 0.651 | -0.002 | 0.773 | -0.011 | 0.287 |
|  | Sn | -0.004 | 0.805 | -0.017 | 0.051 | -0.003 | 0.826 |
|  | Sb | 0.049 | 0.095 | -0.010 | 0.541 | -0.046 | 0.081 |
|  | Cs | -0.001 | 0.982 | -0.011 | 0.438 | -0.049 | 0.045 |
|  | Ba | 0.009 | 0.129 | -0.011 | 0.072 | -0.008 | 0.601 |
|  | W | 0.010 | 0.412 | -0.001 | 0.865 | 0.012 | 0.611 |
|  | Hg | -0.010 | 0.555 | -0.012 | 0.150 | 0.001 | 0.960 |
|  | Tl | 0.011 | 0.621 | -0.028 | 0.044 | -0.017 | 0.524 |
|  | Pb | 0.021 | 0.118 | -0.015 | 0.106 | -0.014 | 0.380 |
|  | Fe | 0.003 | 0.871 | 0.002 | 0.863 | -0.054 | 0.020 |
|  | Co | 0.006 | 0.782 | -0.016 | 0.362 | -0.021 | 0.535 |
|  | Cu | -0.012 | 0.648 | -0.013 | 0.422 | -0.037 | 0.300 |
|  | Zn | 0.058 | 0.150 | -0.017 | 0.479 | -0.084 | 0.020 |
|  | As | 0.004 | 0.311 | -0.005 | 0.206 | -0.006 | 0.509 |
|  | Se | 0.040 | 0.343 | 0.028 | 0.203 | -0.029 | 0.455 |
|  | Rb | -0.001 | 0.968 | -0.015 | 0.370 | -0.025 | 0.393 |
|  | Sr | 0.057 | 0.107 | -0.021 | 0.312 | 0.006 | 0.893 |
|  | Mo | 0.017 | 0.318 | 0.006 | 0.628 | 0.034 | 0.186 |
|  | La | -0.003 | 0.806 | 0.009 | 0.297 | 0.007 | 0.638 |
|  | Ce | 0.019 | 0.304 | 0.000 | 0.974 | -0.015 | 0.441 |
|  | Pr | -0.003 | 0.807 | 0.002 | 0.798 | -0.006 | 0.623 |
|  | Th | 0.002 | 0.873 | 0.004 | 0.589 | -0.002 | 0.875 |
|  | U | 0.007 | 0.589 | -0.002 | 0.785 | -0.002 | 0.841 |

**Table S12 Adjusted associations of ln-transformed metals with alpha diversity, stratified by preterm(≥37 is considered non-preterm, < 37 and≥32 is preterm，<32 is very preterm).** The effect size estimate expressed in the coef column represents the changes in species relative abundance per unit increase in blood metals. *P* value (pval column) calculated from Generalized linear regression. Models were adjusted for Sex, Antibiotic, Ever Breastfed, Delivery Mode, Birth Weight and Sample Age. Bold*: q < 0.01.

| Method | Metals | Non-preterm=95 | | Preterm =191 | | Very preterm=56 | |
| --- | --- | --- | --- | --- | --- | --- | --- |
|  |  | coef | pval | coef | pval | coef | pval |
| Shannon | Cr | -0.005 | 0.859 | 0.004 | 0.864 | -0.106 | 0.022 |
|  | Mn | 0.185 | 0.203 | 0.111 | 0.167 | -0.206 | 0.314 |
|  | Ni | 0.015 | 0.756 | -0.024 | 0.682 | 0.062 | 0.568 |
|  | Cd | -0.007 | 0.921 | 0.026 | 0.380 | -0.035 | 0.570 |
|  | Sn | -0.100 | 0.313 | -0.013 | 0.815 | 0.102 | 0.420 |
|  | Sb | 0.212 | 0.202 | 0.008 | 0.921 | -0.461 | 0.008 |
|  | Cs | -0.084 | 0.575 | -0.080 | 0.295 | -0.382 | 0.015 |
|  | Ba | 0.064 | 0.099 | 0.008 | 0.809 | 0.055 | 0.569 |
|  | W | 0.056 | 0.398 | -0.038 | 0.399 | -0.076 | 0.624 |
|  | Hg | -0.061 | 0.504 | 0.029 | 0.556 | -0.025 | 0.810 |
|  | Tl | -0.001 | 0.994 | -0.197 | **0.007*** | -0.199 | 0.224 |
|  | Pb | 0.118 | 0.151 | -0.028 | 0.603 | -0.045 | 0.654 |
|  | Fe | 0.005 | 0.960 | 0.096 | 0.197 | -0.370 | 0.009 |
|  | Co | -0.036 | 0.755 | -0.073 | 0.451 | -0.235 | 0.269 |
|  | Cu | -0.156 | 0.334 | -0.370 | **0.0004*** | -0.326 | 0.178 |
|  | Zn | 0.318 | 0.155 | 0.016 | 0.899 | -0.515 | 0.026 |
|  | As | 0.029 | 0.277 | 0.040 | 0.057 | 0.103 | 0.100 |
|  | Se | 0.177 | 0.557 | 0.071 | 0.602 | -0.413 | 0.158 |
|  | Rb | -0.085 | 0.629 | 0.028 | 0.754 | -0.216 | 0.257 |
|  | Sr | 0.265 | 0.176 | -0.225 | 0.046 | 0.164 | 0.609 |
|  | Mo | 0.048 | 0.657 | 0.079 | 0.260 | 0.241 | 0.136 |
|  | La | -0.045 | 0.499 | -0.023 | 0.620 | -0.025 | 0.787 |
|  | Ce | 0.050 | 0.612 | -0.016 | 0.782 | -0.077 | 0.526 |
|  | Pr | -0.033 | 0.606 | -0.034 | 0.353 | -0.056 | 0.456 |
|  | Th | 0.001 | 0.990 | -0.031 | 0.428 | -0.041 | 0.610 |
|  | U | -0.021 | 0.762 | -0.021 | 0.529 | -0.049 | 0.527 |
| Simpson | Cr | 0.002 | 0.813 | 0.001 | 0.919 | -0.021 | 0.179 |
|  | Mn | 0.069 | 0.244 | 0.055 | 0.077 | -0.118 | 0.074 |
|  | Ni | 0.003 | 0.868 | -0.010 | 0.660 | -0.006 | 0.874 |
|  | Cd | -0.013 | 0.662 | 0.013 | 0.252 | -0.015 | 0.466 |
|  | Sn | -0.047 | 0.249 | -0.006 | 0.767 | 0.023 | 0.572 |
|  | Sb | 0.082 | 0.226 | 0.005 | 0.866 | -0.109 | 0.059 |
|  | Cs | -0.011 | 0.861 | -0.038 | 0.198 | -0.150 | **0.003*** |
|  | Ba | 0.024 | 0.129 | 0.004 | 0.777 | 0.014 | 0.661 |
|  | W | 0.018 | 0.496 | -0.016 | 0.358 | -0.006 | 0.898 |
|  | Hg | -0.028 | 0.444 | -0.001 | 0.962 | 0.012 | 0.722 |
|  | Tl | -0.004 | 0.929 | -0.087 | **0.002*** | -0.063 | 0.242 |
|  | Pb | 0.036 | 0.288 | -0.004 | 0.856 | -0.033 | 0.320 |
|  | Fe | 0.008 | 0.828 | 0.042 | 0.139 | -0.106 | 0.024 |
|  | Co | 0.002 | 0.962 | -0.035 | 0.338 | -0.043 | 0.540 |
|  | Cu | -0.039 | 0.552 | -0.131 | **0.001*** | 0.011 | 0.885 |
|  | Zn | 0.105 | 0.247 | 0.025 | 0.605 | -0.227 | **0.002*** |
|  | As | 0.012 | 0.267 | 0.011 | 0.191 | 0.017 | 0.408 |
|  | Se | 0.043 | 0.723 | 0.008 | 0.880 | -0.142 | 0.137 |
|  | Rb | -0.027 | 0.707 | -0.004 | 0.899 | -0.094 | 0.129 |
|  | Sr | 0.112 | 0.161 | -0.103 | 0.017 | 0.131 | 0.205 |
|  | Mo | 0.029 | 0.512 | 0.026 | 0.332 | 0.078 | 0.141 |
|  | La | -0.011 | 0.673 | -0.002 | 0.895 | -0.001 | 0.966 |
|  | Ce | 0.024 | 0.549 | -0.006 | 0.788 | -0.012 | 0.769 |
|  | Pr | -0.008 | 0.747 | -0.010 | 0.483 | -0.014 | 0.560 |
|  | Th | 0.009 | 0.770 | -0.013 | 0.373 | -0.006 | 0.810 |
|  | U | -0.013 | 0.634 | -0.007 | 0.578 | -0.008 | 0.762 |
| Chao | Cr | -0.414 | 0.644 | -0.872 | 0.190 | -3.555 | **0.007*** |
|  | Mn | 5.042 | 0.324 | 3.398 | 0.142 | 3.730 | 0.526 |
|  | Ni | 2.056 | 0.224 | -2.539 | 0.132 | 2.106 | 0.495 |
|  | Cd | -0.460 | 0.854 | 1.032 | 0.229 | 2.178 | 0.221 |
|  | Sn | -1.021 | 0.770 | -0.658 | 0.681 | 5.108 | 0.158 |
|  | Sb | -4.632 | 0.429 | -1.911 | 0.431 | -17.943 | **0.00019*** |
|  | Cs | -1.926 | 0.713 | -2.289 | 0.297 | -4.829 | 0.295 |
|  | Ba | 1.813 | 0.184 | 2.361 | **0.011*** | 3.352 | 0.223 |
|  | W | -1.012 | 0.666 | -2.727 | 0.035 | -7.478 | 0.088 |
|  | Hg | -1.978 | 0.534 | 1.069 | 0.451 | -2.444 | 0.407 |
|  | Tl | -9.844 | 0.015 | -2.568 | 0.226 | -4.725 | 0.316 |
|  | Pb | 3.880 | 0.179 | 1.824 | 0.230 | 0.543 | 0.852 |
|  | Fe | -1.295 | 0.690 | 0.554 | 0.795 | -6.053 | 0.147 |
|  | Co | -7.921 | 0.049 | -3.126 | 0.258 | -10.171 | 0.092 |
|  | Cu | -3.677 | 0.517 | -8.254 | **0.006*** | -13.234 | 0.054 |
|  | Zn | 5.990 | 0.447 | 1.429 | 0.697 | -7.541 | 0.266 |
|  | As | 1.820 | 0.051 | 2.268 | **0.00013*** | 5.028 | **0.004*** |
|  | Se | -11.484 | 0.277 | -0.272 | 0.945 | -14.059 | 0.093 |
|  | Rb | 0.890 | 0.886 | 2.066 | 0.425 | -5.049 | 0.357 |
|  | Sr | 4.804 | 0.487 | -4.306 | 0.185 | -3.923 | 0.669 |
|  | Mo | -6.695 | 0.078 | 0.381 | 0.850 | 1.434 | 0.760 |
|  | La | -4.656 | 0.043 | -2.569 | 0.055 | -2.571 | 0.336 |
|  | Ce | -4.196 | 0.226 | -0.576 | 0.733 | -1.547 | 0.658 |
|  | Pr | -5.862 | 0.008 | -1.640 | 0.116 | -1.391 | 0.522 |
|  | Th | -2.986 | 0.246 | -1.081 | 0.331 | -2.788 | 0.221 |
|  | U | -7.329 | **0.002*** | -0.313 | 0.748 | -3.710 | 0.088 |
| Pielou | Cr | 0.001 | 0.875 | 0.002 | 0.594 | -0.012 | 0.127 |
|  | Mn | 0.027 | 0.343 | 0.017 | 0.230 | -0.056 | 0.100 |
|  | Ni | -0.001 | 0.951 | -0.002 | 0.841 | 0.004 | 0.833 |
|  | Cd | -0.001 | 0.935 | 0.003 | 0.521 | -0.013 | 0.198 |
|  | Sn | -0.022 | 0.253 | -0.001 | 0.955 | 0.007 | 0.751 |
|  | Sb | 0.046 | 0.151 | 0.004 | 0.772 | -0.054 | 0.068 |
|  | Cs | -0.005 | 0.857 | -0.012 | 0.399 | -0.064 | 0.015 |
|  | Ba | 0.009 | 0.230 | -0.003 | 0.620 | 0.000 | 0.990 |
|  | W | 0.014 | 0.280 | -0.003 | 0.730 | 0.002 | 0.924 |
|  | Hg | -0.008 | 0.648 | 0.003 | 0.725 | 0.003 | 0.882 |
|  | Tl | 0.015 | 0.515 | -0.035 | **0.007*** | -0.026 | 0.343 |
|  | Pb | 0.015 | 0.330 | -0.008 | 0.400 | -0.014 | 0.402 |
|  | Fe | 0.007 | 0.715 | 0.019 | 0.154 | -0.060 | 0.013 |
|  | Co | 0.010 | 0.669 | -0.008 | 0.629 | -0.024 | 0.494 |
|  | Cu | -0.020 | 0.513 | -0.051 | **0.006*** | -0.025 | 0.534 |
|  | Zn | 0.053 | 0.216 | 0.004 | 0.860 | -0.097 | 0.012 |
|  | As | 0.002 | 0.643 | 0.003 | 0.425 | 0.008 | 0.443 |
|  | Se | 0.061 | 0.293 | 0.019 | 0.449 | -0.053 | 0.286 |
|  | Rb | -0.012 | 0.722 | -0.003 | 0.853 | -0.035 | 0.271 |
|  | Sr | 0.052 | 0.165 | -0.037 | 0.069 | 0.035 | 0.517 |
|  | Mo | 0.019 | 0.377 | 0.016 | 0.214 | 0.041 | 0.131 |
|  | La | 0.001 | 0.921 | 0.002 | 0.815 | 0.001 | 0.965 |
|  | Ce | 0.019 | 0.309 | 0.000 | 0.964 | -0.012 | 0.547 |
|  | Pr | 0.004 | 0.753 | -0.002 | 0.730 | -0.008 | 0.519 |
|  | Th | 0.007 | 0.599 | -0.002 | 0.755 | -0.003 | 0.804 |
|  | U | 0.006 | 0.634 | -0.002 | 0.713 | -0.001 | 0.943 |

**Table S13 Associations between ln-transformed metals and beta diversity in unadjusted models, stratified by preterm(≥37 is considered non-preterm, < 37 and≥32 is preterm，<32 is very preterm).** *P values* (pval columns) were calculated from adonis models. (R^2^) is the fraction of variance explained. Bold*: q < 0.01.

| **Metals** | Non-preterm=95 | | Preterm =191 | | Very preterm=56 | |
| --- | --- | --- | --- | --- | --- | --- |
|  | pval | R^2^ | pval | R^2^ | pval | R^2^ |
| Cr | 0.758 | 0.007152654 | 0.61 | 0.004181712 | 0.327 | 0.019882161 |
| Mn | 0.812 | 0.007152654 | 0.154 | 0.008171249 | 0.643 | 0.014384954 |
| Ni | 0.579 | 0.009345213 | 0.067 | 0.009260652 | 0.803 | 0.011029684 |
| Cd | 0.67 | 0.008574166 | 0.303 | 0.005830096 | 0.331 | 0.020466552 |
| Sn | 0.331 | 0.011893102 | **0.006*** | 0.015810433 | 0.045 | 0.034353993 |
| Sb | 0.402 | 0.011002736 | **0.006*** | 0.015849314 | 0.78 | 0.011838107 |
| Cs | 0.809 | 0.006966209 | 0.068 | 0.009346993 | 0.204 | 0.023235302 |
| Ba | 0.347 | 0.01140335 | 0.244 | 0.006526665 | 0.327 | 0.020435931 |
| W | 0.508 | 0.010160073 | 0.962 | 0.002076225 | 0.758 | 0.012430802 |
| Hg | 0.431 | 0.010673199 | 0.183 | 0.007386059 | 0.162 | 0.025597816 |
| Tl | 0.47 | 0.010445246 | 0.249 | 0.006535937 | 0.698 | 0.013295046 |
| Pb | 0.803 | 0.0067846 | 0.514 | 0.004614352 | 0.274 | 0.020986346 |
| Fe | 0.873 | 0.006102881 | 0.739 | 0.003540776 | 0.851 | 0.010443504 |
| Co | 0.282 | 0.012379701 | 0.795 | 0.003215613 | 0.54 | 0.016073158 |
| Cu | 0.621 | 0.008883871 | 0.093 | 0.008537642 | 0.286 | 0.021089172 |
| Zn | 0.469 | 0.009874656 | 0.892 | 0.002826983 | 0.256 | 0.022604078 |
| As | 0.617 | 0.008902241 | 0.044 | 0.010625237 | 0.435 | 0.018180304 |
| Se | 0.178 | 0.014315478 | 0.197 | 0.006780824 | 0.14 | 0.026093837 |
| Rb | 0.043 | 0.019830614 | 0.17 | 0.007492158 | 0.805 | 0.010798849 |
| Sr | 0.103 | 0.016265078 | 0.049 | 0.009941379 | 0.513 | 0.015789411 |
| Mo | 0.421 | 0.010523096 | 0.9 | 0.002618845 | 0.068 | 0.031291543 |
| La | 0.402 | 0.010552418 | 0.355 | 0.005484224 | 0.761 | 0.011909595 |
| Ce | 0.335 | 0.01180222 | 0.321 | 0.005836742 | 0.951 | 0.007839907 |
| Pr | 0.402 | 0.010951894 | 0.317 | 0.006100843 | 0.986 | 0.005461423 |
| Th | 0.543 | 0.009344586 | 0.146 | 0.007518006 | 0.955 | 0.007766691 |
| U | 0.621 | 0.008740618 | 0.085 | 0.008828552 | 0.995 | 0.005175604 |

**Table S14 Associations between ln-transformed metals and beta diversity in adjusted models, stratified by preterm(≥37 is considered non-preterm, < 37 and≥32 is preterm，<32 is very preterm).** *P values* (pval columns) were calculated from adonis models. (R^2^) is the fraction of variance explained, Models were adjusted for Sex, Antibiotic, Ever Breastfed, Delivery Mode, Birth Weight and Sample Age. Bold*: q < 0.01.

| **Metals** | Non-preterm=95 | | Preterm =191 | | Very preterm=56 | |
| --- | --- | --- | --- | --- | --- | --- |
|  | pval | R^2^ | pval | R^2^ | pval | R^2^ |
| Cr | 0.82 | 0.00652325 | 0.679 | 0.003926698 | 0.046 | 0.034653516 |
| Mn | 0.608 | 0.008362081 | 0.078 | 0.009456318 | 0.533 | 0.015917367 |
| Ni | 0.481 | 0.009640641 | 0.515 | 0.004652003 | 0.597 | 0.015163583 |
| Cd | 0.537 | 0.0093275 | 0.32 | 0.00598275 | 0.415 | 0.018625502 |
| Sn | 0.661 | 0.008021304 | 0.305 | 0.005885146 | 0.353 | 0.019534313 |
| Sb | 0.938 | 0.004889937 | 0.011 | 0.013729256 | 0.353 | 0.019831721 |
| Cs | 0.11 | 0.015137966 | 0.154 | 0.007788955 | 0.214 | 0.024594139 |
| Ba | 0.071 | 0.016823564 | 0.175 | 0.007236576 | 0.199 | 0.023776862 |
| W | 0.411 | 0.010470108 | 0.92 | 0.002419377 | 0.785 | 0.011535073 |
| Hg | 0.672 | 0.007790154 | 0.36 | 0.005535622 | 0.254 | 0.022493023 |
| Tl | 0.483 | 0.009430773 | 0.274 | 0.006058359 | 0.581 | 0.014951634 |
| Pb | 0.568 | 0.0088006 | 0.477 | 0.004680225 | 0.137 | 0.025845016 |
| Fe | 0.753 | 0.007067196 | 0.923 | 0.00250581 | 0.659 | 0.013850766 |
| Co | 0.338 | 0.011014972 | 0.927 | 0.002416928 | 0.253 | 0.022202948 |
| Cu | 0.229 | 0.012481078 | 0.051 | 0.009679066 | 0.37 | 0.018984005 |
| Zn | 0.672 | 0.008069645 | 0.807 | 0.003127059 | 0.212 | 0.02429323 |
| As | 0.087 | 0.016187091 | 0.008 | 0.014953986 | 0.286 | 0.021660811 |
| Se | 0.773 | 0.007012358 | 0.78 | 0.003281618 | 0.457 | 0.017315049 |
| Rb | 0.125 | 0.015309311 | 0.188 | 0.00712477 | 0.512 | 0.016217904 |
| Sr | 0.341 | 0.011104963 | 0.088 | 0.008502806 | 0.641 | 0.014539371 |
| Mo | 0.319 | 0.011341041 | 0.906 | 0.002666934 | 0.277 | 0.021915343 |
| La | 0.097 | 0.015543133 | 0.344 | 0.005490678 | 0.682 | 0.01357015 |
| Ce | 0.381 | 0.010435484 | 0.444 | 0.005075743 | 0.886 | 0.009466576 |
| Pr | 0.396 | 0.010501358 | 0.301 | 0.0060017 | 0.991 | 0.005597997 |
| Th | 0.524 | 0.008839784 | 0.076 | 0.009281824 | 0.974 | 0.007108866 |
| U | 0.991 | 0.003466243 | 0.093 | 0.008375374 | 0.996 | 0.004897084 |

**Table S15 Non preterm(N=95) -- Adjusted taxa associations from 16S rRNA gene amplicon sequencing.** The effect size estimate expressed in the coef column represents the changes in species relative abundance (ln transformed) per unit increase in blood metals. Associations were analyzed using MaAsLin2. False discovery rate (FDR) adjusted *q* values are in the last column (qval). Models were adjusted for Sex, Antibiotic, Ever Breastfed, Delivery Mode, Birth Weight and Sample Age

| Metals | Phylum | genus | Coef | SE | p-Value | FDR_q-Value |
| --- | --- | --- | --- | --- | --- | --- |
| Tl | *Acidobacteriota* |  | -0.433644853 | 0.129240213 | 0.001176352 | 0.082344673 |
| U | *Acidobacteriota* |  | -0.612449328 | 0.128780691 | 7.78E-06 | 0.000544507 |
| U | *Chloroflexi* |  | -0.630300128 | 0.150803636 | 6.93E-05 | 0.002425676 |
| Ba | *Proteobacteria* | *Ralstonia* | -0.313765729 | 0.092206175 | 0.001009814 | 0.040998454 |
| Cu | *Proteobacteria* | *Ralstonia* | 0.341011491 | 0.088915149 | 0.000236878 | 0.024043145 |
| Zn | *Firmicutes* | *Robinsoniella* | 0.727644134 | 0.213704983 | 0.001003224 | 0.050913629 |
| Pr | *Proteobacteria* | *Acinetobacter* | -0.781810226 | 0.233253908 | 0.001189904 | 0.048310084 |

**Table S16 preterm (N=191) -- Adjusted taxa associations from 16S rRNA gene amplicon sequencing.** The effect size estimate expressed in the coef column represents the changes in species relative abundance (ln transformed) per unit increase in blood metals. Associations were analyzed using MaAsLin2. False discovery rate (FDR) adjusted *q* values are in the last column (qval). Models were adjusted for Sex, Antibiotic, Ever Breastfed, Delivery Mode, Birth Weight and Sample Age

| Metals | Phylum | genus | Coef | SE | p-Value | FDR_q-Value |
| --- | --- | --- | --- | --- | --- | --- |
| Cs | *Gemmatimonadota* |  | 0.237417674 | 0.076031647 | 0.002082364 | 0.045812002 |
| Tl | *Gemmatimonadota* |  | 0.266447564 | 0.072842148 | 0.000331917 | 0.021906543 |
| Se | *Gemmatimonadota* |  | 0.225758625 | 0.076778528 | 0.003698815 | 0.081373919 |
| Cr | *Firmicutes* | *Lactococcus* | -0.479159421 | 0.141323652 | 0.000854625 | 0.041876607 |
| Mn | *Firmicutes* | *Enterococcus* | -0.792092525 | 0.228952879 | 0.000672976 | 0.032975818 |
| Mn | *Proteobacteria* | *Burkholderia.Caballeronia.Paraburkholderia* | -0.405637185 | 0.127717398 | 0.001752313 | 0.061330939 |
| Ba | *Proteobacteria* | *Acinetobacter* | 0.77485609 | 0.241178017 | 0.001553387 | 0.063429952 |
| Ba | *Firmicutes* | *Lactobacillus* | 0.526040213 | 0.166465213 | 0.001846055 | 0.064611925 |
| Pb | *Proteobacteria* | *Ralstonia* | -0.446666038 | 0.096917657 | 7.58E-06 | 0.000928506 |
| Pb | *Acidobacteriota* | *Cutibacterium* | -0.556536581 | 0.181148482 | 0.002448799 | 0.085707962 |
| Cu | *Acidobacteriota* | *Cutibacterium* | -0.866892299 | 0.204632428 | 3.60E-05 | 0.002367116 |
| Cu | *Firmicutes* | *Veillonella* | -1.195281417 | 0.367384495 | 0.001357672 | 0.036958851 |
| As | *Proteobacteria* | *Burkholderia.Caballeronia.Paraburkholderia* | -0.589943206 | 0.135461939 | 2.21E-05 | 0.00270924 |
| As | *Proteobacteria* | *Ralstonia* | -0.405107381 | 0.103838127 | 0.00013427 | 0.008224029 |
| As | *Firmicutes* | *Veillonella* | 1.187418537 | 0.334169721 | 0.000483936 | 0.019760708 |
| As | *Proteobacteria* | *Acinetobacter* | 0.854784545 | 0.256179592 | 0.001026892 | 0.027954272 |
| As | *Firmicutes* | *Enterococcus* | -0.750307547 | 0.250277932 | 0.003096321 | 0.052475165 |
| La | *Proteobacteria* | *Burkholderia.Caballeronia.Paraburkholderia* | 0.404013444 | 0.126947215 | 0.001715609 | 0.070054023 |
| Th | *Acidobacteriota* | *Rothia* | -1.02907319 | 0.239906832 | 2.90E-05 | 0.00354779 |
| Th | *Proteobacteria* | *Burkholderia.Caballeronia.Paraburkholderia* | 0.431677845 | 0.124752029 | 0.00067144 | 0.027417124 |
| Th | *Proteobacteria* | *Sphingomonas* | -0.262875687 | 0.08073382 | 0.001346027 | 0.036641839 |
| Th | *Proteobacteria* | *Acinetobacter* | -0.681748848 | 0.233372852 | 0.003924903 | 0.087418301 |

**Table S17 Very preterm (N=56) -- Adjusted taxa associations from 16S rRNA gene amplicon sequencing.** The effect size estimate expressed in the coef column represents the changes in species relative abundance (ln transformed) per unit increase in blood metals. Associations were analyzed using MaAsLin2. False discovery rate (FDR) adjusted *q* values are in the last column (qval). Models were adjusted for Sex, Antibiotic, Ever Breastfed, Delivery Mode, Birth Weight and Sample Age

| Metals | Phylum | genus | Coef | SE | p-Value | FDR_q-Value |
| --- | --- | --- | --- | --- | --- | --- |
| Se | *Verrucomicrobiota* |  | -1.60146162 | 0.42020345 | 0.000393901 | 0.024815752 |
| Sn | *Proteobacteria* | *Stenotrophomonas* | 1.50066944 | 0.379598114 | 0.000252626 | 0.070735338 |
| Sb | *Acidobacteriota* | *Cutibacterium* | -1.218225002 | 0.328829907 | 0.000546774 | 0.051032251 |
| Sb | *Firmicutes* | *Exiguobacterium* | -0.70706269 | 0.18817992 | 0.000465133 | 0.051032251 |
| Sb | *Firmicutes* | *Staphylococcus* | -1.721564416 | 0.503856182 | 0.001299405 | 0.090958378 |


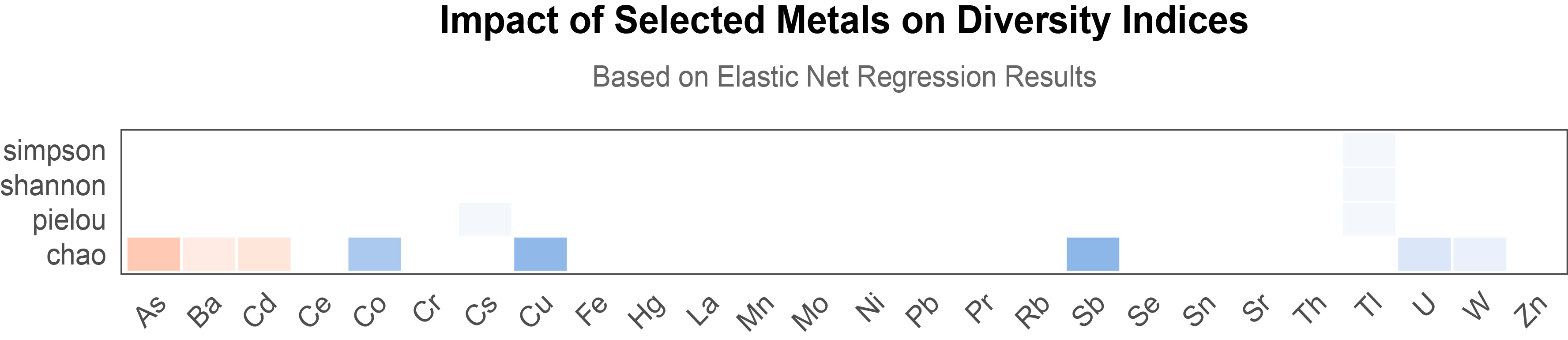


**Figure S1 Elastic net-selected metals significantly associated with alpha-diversity indices,** with orange/blue bars denoting positive/negative associations and shading intensity representing effect magnitude.
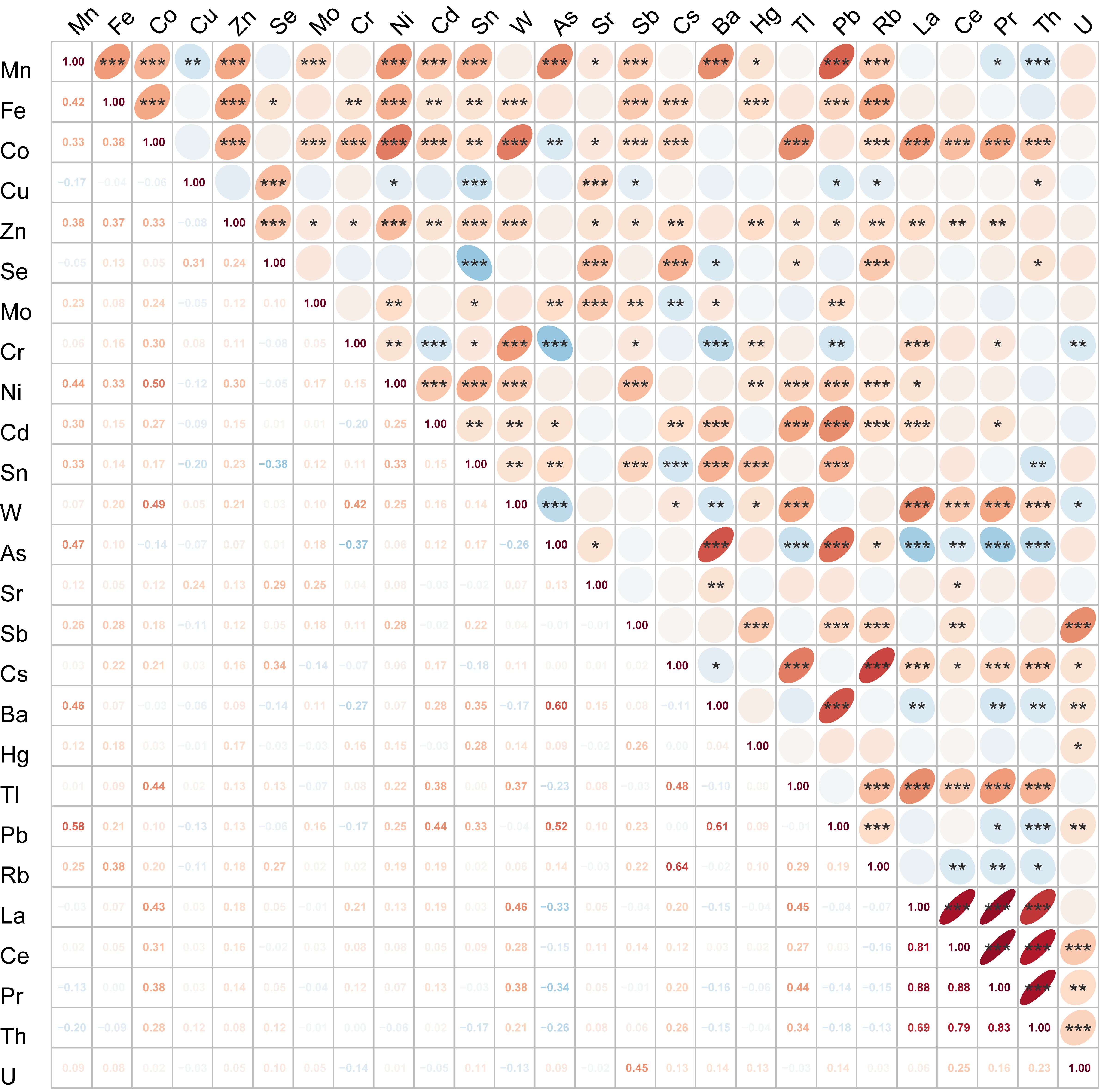


**Figure S2 Associations between ln-transformed metals and alpha diversity**





**Figure S3 Associations between ln-transformed metals and 3 taxa (BKMR).** Models were adjusted for Preterm, Sex, Antibiotic, Ever Breastfed, Delivery Mode, Birth Weight and Sample Age

**

**

**Figure S4. Metal-related metabolic pathways of the gut microbiota.** (A) Pathways associated with manganese. (B) Pathways associated with arsenic.
