# Supplementary figures and images for: Association between infants’ serum levels of 26 metals and gut microbiota: a hospital-based cross-sectional study in China
Source: Front Microbiol. 2025 Dec 11;16:1669475. doi: 10.3389/fmicb.2025.1669475 (PMC12740241; doi:10.3389/fmicb.2025.1669475)

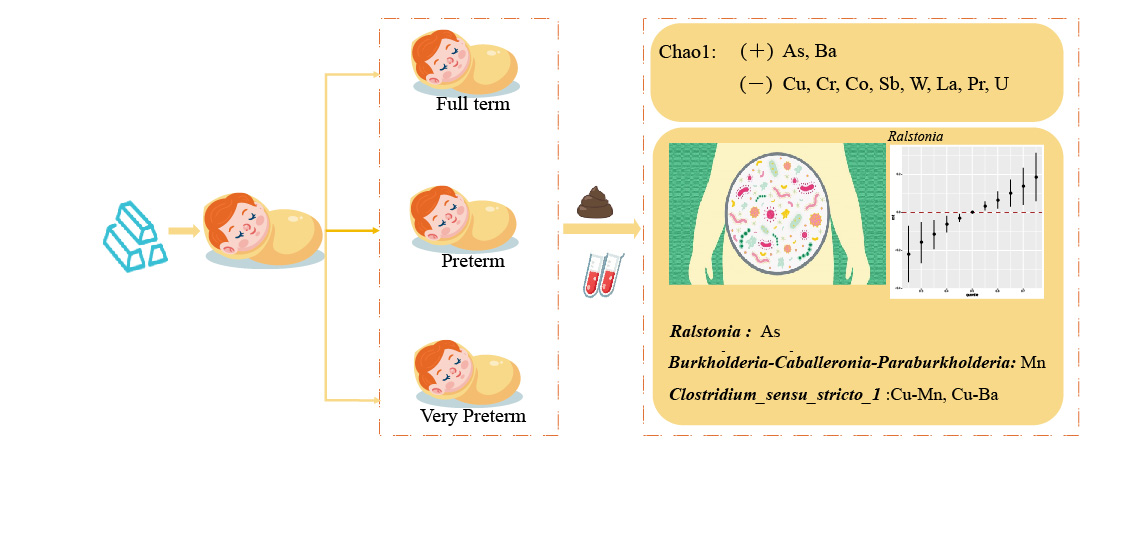

Supplement: Supplementary file 2 [file Image_1.jpeg]
